# Novel insights in the pathomechanism of Brugada syndrome and fever‐related type 1 ECG changes in a preclinical study using human‐induced pluripotent stem cell‐derived cardiomyocytes

**DOI:** 10.1002/ctm2.1130

**Published:** 2023-03-07

**Authors:** Yingrui Li, Hendrik Dinkel, Dalia Pakalniskyte, Alexandra Viktoria Busley, Lukas Cyganek, Rujia Zhong, Feng Zhang, Qiang Xu, Lasse Maywald, Assem Aweimer, Mengying Huang, Zhenxing Liao, Zenghui Meng, Chen Yan, Timo Prädel, Lena Rose, Alexander Moscu‐Gregor, Alyssa Hohn, Zhen Yang, Lin Qiao, Andreas Mügge, Xiaobo Zhou, Ibrahim Akin, Ibrahim El‐Battrawy

**Affiliations:** ^1^ First Department of Medicine Faculty of Medicine University Medical Centre Mannheim (UMM) Heidelberg University Mannheim Germany; ^2^ DZHK (German Center for Cardiovascular Research) Partner Site Heidelberg‐Mannheim and Göttingen Mannheim Germany; ^3^ Stem Cell Unit Clinic for Cardiology and Pneumology University Medical Center Göttingen Göttingen Germany; ^4^ Key Laboratory of Medical Electrophysiology of Ministry of Education and Medical Electrophysiological Key Laboratory of Sichuan Province Institute of Cardiovascular Research Southwest Medical University Luzhou China; ^5^ Department of Cardiology and Angiology Bergmannsheil University Hospitals Ruhr University of Bochum Bochum Germany; ^6^ Center for Human Genetics and Laboratory Medicine Martinsried Germany

**Keywords:** autophaghy, Brugada syndrome, cardiac arrest, induced pluripotent stem cells, inflammation, sudden cardiac death

## Abstract

**Background:**

Brugada syndrome (BrS) is causing sudden cardiac death (SCD) mainly at young age. Studying the underlying mechanisms associated with BrS type I electrocardiogram (ECG) changes in the presence of fever and roles of autophagy for BrS remains lacking.

**Objectives:**

We sought to study the pathogenic role of an *SCN5A* gene variant for BrS with fever‐induced type 1 ECG phenotype. In addition, we studied the role of inflammation and autophagy in the pathomechanism of BrS.

**Methods:**

Human‐induced pluripotent stem cell (hiPSC) lines from a BrS patient harboring a pathogenic variant (c.3148G>A/p. Ala1050Thr) in *SCN5A* and two healthy donors (non‐BrS) and a CRISPR/Cas9 site‐corrected cell line (BrS‐corr) were differentiated into cardiomyocytes (hiPSC‐CMs) for the study.

**Results:**

Reductions of Na_v_1.5 expression, peak sodium channel current (I_Na_) and upstroke velocity (V_max_) of action potentials with an increase in arrhythmic events were detected in BrS compared to non‐BrS and BrS‐corr cells. Increasing the cell culture temperature from 37 to 40°C (fever‐like state) exacerbated the phenotypic changes in BrS cells. The fever‐effects were enhanced by protein kinase A (PKA) inhibitor but reversed by PKA activator. Lipopolysaccharides (LPS) but not increased temperature up to 40°C enhanced the autophagy level in BrS‐hiPSC‐CMs by increasing reactive oxidative species and inhibiting PI3K/AKT signalling, and hence exacerbated the phenotypic changes. LPS enhanced high temperature‐related effect on peak I_Na_ shown in BrS hiPSC‐CMs. Effects of LPS and high temperature were not detected in non‐BrS cells.

**Conclusions:**

The study demonstrated that the SCN5A variant (c.3148G>A/p.Ala1050Thr) caused loss‐of‐function of sodium channels and increased the channel sensitivity to high temperature and LPS challenge in hiPSC‐CMs from a BrS cell line with this variant but not in two non‐BrS hiPSC‐CM lines. The results suggest that LPS may exacerbate BrS phenotype via enhancing autophagy, whereas fever may exacerbate BrS phenotype via inhibiting PKA‐signalling in BrS cardiomyocytes with but probably not limited to this variant.

## Graphical abstract

1. SCN5A variant (c.3148G>A/p.Ala1050Thr) causes loss‐of‐function of sodium channels in human‐induced pluripotent stem cell‐derived cardiomyocytes (hiPSC‐CMs) from BrS patient.
2. SCN5A variant (c.3148G>A/p.Ala1050Thr) increases the channel sensitivity to hyperthermia and LPS challenge in hiPSC‐CMs from BrS patient.
3. Autophagy and PKA pathway mediate the effect of LPS and hyperthermia on hiPSC‐CMs from BrS patient with SCN5A variant (c.3148G>A/p.Ala1050Thr), respectively.

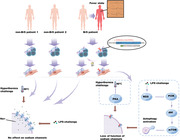

## 1 INTRODUCTION

Brugada syndrome (BrS), a rare inherited channelopathy, is linked to sudden cardiac death (SCD). BrS type 1 electrocardiogram (ECG) could be unmasked by fever, sodium channel blockers and increased parasympathetic state.^1^ It has been suggested to avoid fever, infection and/or sodium channel inhibiting drugs, since they may increase the risk of ventricular tachyarrhythmias.

More than 50 gene mutations have been associated with BrS.^2^, ^3^ But nevertheless, 20%–30% of BrS cases are related to mutations or variants in the *SCN5A* gene (encoding the ion channel Na_v_1.5). Among different variants in the *SCN5A* gene, the pathogenic role of each variant remains to be experimentally validated.^4^ One important limitation is that the role of a bevy of variants has been studied using animal models or heterologous expression systems, which might differ from human cardiomyocytes. Recently published data showed that genetics is associated with cellular and clinical phenotype severity, which has been studied in BrS patients carrying *SCN5A* mutations and undergoing electrophysiology studies and epicardial mapping.^5^, ^6^

Despite advances in BrS, the underlying pathomechanism remains unclear. Even more, the causal association between fever and/or inflammation and BrS phenotype has not been studied yet in human cardiomyocytes. Recent study indicated that fever mediated the formation of the autophagosome.^7^ Autophagy is an intracellular process that can engulf targeted cytoplasmic proteins or organelles by a double membrane‐bound vesicle called autophagosome that fuses with lysosomes for degradation.^8^ Autophagy process included initiation with generating isolated membrane, membrane elongation, membrane closure, fusion with lysosomes and targeted substrates degradation.^9^ To date, more and more research has explored the effect of autophagy on ion channels because of its function to regulate cell homeostasis and survival. Cardiomyocytes from atrial fibrillation patients and a rabbit model of atrial rapid pacing both showed increased autophagy level, and L‐type calcium current (I_CaL_) reduction after overexpressing ATG7 in atrial cardiomyocytes.^10^ In another study, Ca^2+^‐activated chloride channel in basilar artery smooth muscle cells of renohypertesive rats could be down‐regulated by autophagy‐mediated TMEM16A degradation.^11^ However, the effect of autophagy on BrS remains unknown.

Inflammation is one of the most common pathological states seen in human and is usually the culprit of fever. Recent studies displayed the important role of inflammation in pathogenesis of BrS.^12^, ^13^ A case report study showed that two well‐characterized BrS patients with acute cardiac inflammation experienced frequently ventricular fibrillation episodes, which showed a possible cause of inflammation for the occurrence of malignant ventricular arrhythmias in BrS.^14^ However, the exact roles and pathological mechanism of inflammation in BrS remain unclear.

This study was designed to investigate the pathogenic role of an SCN5A gene variant for BrS with fever‐induced type 1 ECG phenotype and explore possible impact of inflammation and autophagy in the pathomechanism of BrS.

## 2 MATERIALS AND METHODS

*Ethics*. The studies generating and using human‐induced pluripotent stem cells (hiPSCs) and the stem cell derived cardiomyocytes (hiPSC‐CMs) were approved by the Medical Ethics Committee II of the University of Heidelberg on 01/05/2010 and 04/12/2012 (2009‐350N‐MA and 2018–565N‐MA). The Ethics Committee of the University Medical Center Göttingen (10/9/15) gave approval on 10/09/2015. The Declaration of Helsinki 1975 of the World Medical Association in its revised version of 2013 served as the basis for the study. Skin biopsies of heart‐healthy donors and BrS patients were performed after obtaining written informed consent.

A detailed description of methods used for the study is provided in the online supplementary information. Briefly, single nucleotide polymorphism c.3148G>A (p. Ala1050Thr) was identified in *SCN5A* by Sanger‐Setup. Reprogramming of the BrS patient dermal fibroblasts into hiPSC lines isBrSe1.1/10/20 (UMGi127‐A clone 1, clone 10, clone 20, here abbreviated as BrS) was performed by using the CytoTune‐iPS 2.0 Sendai Reprogramming Kit according to established protocols, described previously.^15^ The hiPSC cell line isBrsd2.1‐40 (here abbreviated as BrS‐*CANCB2*) was generated from a BrS patient with a *CANCB2* variant (c.428C>T/p.Ser143Phe) by using CytoTune‐iPS 2.0 Sendai Reprogramming Kit. The non‐BrS hiPSC lines ipWT1.1 (UMGi014‐B clone 1, here abbreviated as non‐BrS1) and isWT11.5 (UMGi130‐A clone 5, here abbreviated as non‐BrS2) were generated from dermal fibroblasts and peripheral blood mononuclear cells, respectively, using integration‐free episomal plasmids or integration‐free CytoTune‐iPS 2.0 Sendai virus, respectively, and characterized as previously described.^16^ The single nucleotide polymorphism c.3148G>A (p. Ala1050Thr) in *SCN5A* (Gene ID: 6331) and variant (c.428C>T/p.Ser143Phe) in *CACNB2* (Gene ID: 783) were corrected using CRISPR/Cas9 technology. The BrS, non‐BrS and BrS‐corr hiPSCs were differentiated into cardiomyocytes. Only cardiomyocytes that were at least at day 45 of differentiation were used for the experiments in this study to ensure adequate gene expression and protein biosynthesis of cardiac genes. qPCR, western blot, flow cytometry, calcium imaging, immunofluorescence and patch clamp analyses were performed for the study.

*Statistical analysis*. Statistical analysis of the different measurements was performed using InStat and SigmaPlot. The Kolmogorov‐Smirnov test was used to examine the normal distribution of data. To test for significance, the unpaired Student's *t*‐test was used to compare two independent groups. One‐way analysis of variance (ANOVA) followed with Holm‐Sidak post‐test for multiple comparisons was used for comparing more than two groups. *p*‐Values < .05 were considered statistically significant. The Fisher‐test was applied for comparing categorical variables. The number of cells (immunohistochemistry, patch clamp) and biological replicates (qPCR, Western blot) was described by the letter *n*.

## 3 RESULTS

### 3.1 Characterization of patient‐specific hiPSCs and hiPSC‐CMs from the BrS patient

**FIGURE 1:**
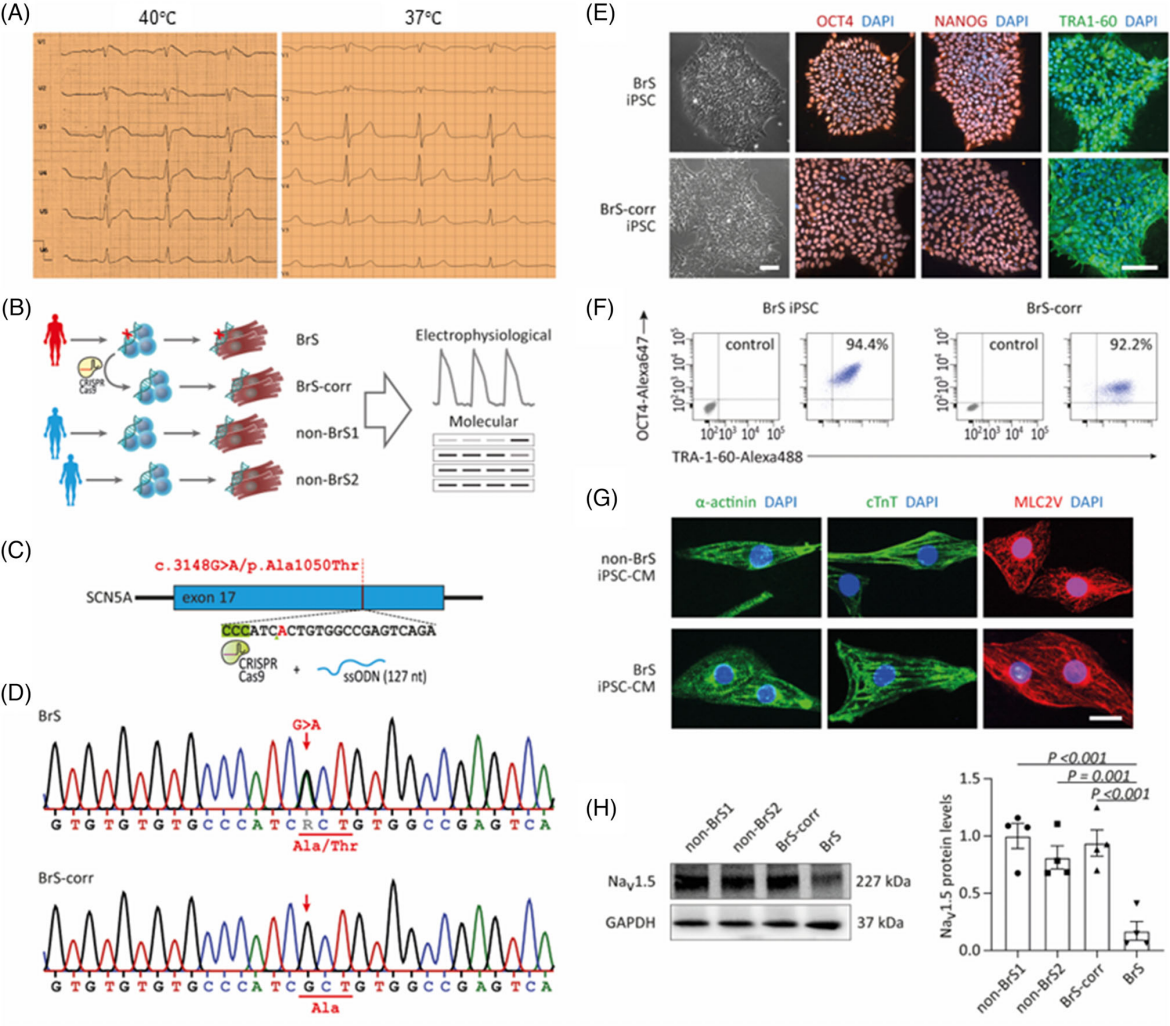
Expression of cardio‐specific markers and pluripotency markers. (A) An electrocardiogram (ECG) of the patient at fever situation presents typical BrS changes (left slide). At physiological temperature, no BrS ECG changes are recorded (right slide). (B) Schematic outline of this study and overview of hiPSC lines used. (C) Representation of CRISPR/cas9‐based gene editing strategy for site‐specific correction of the SNP c.3148G>A/p. Ala1050Thr in SCN5A. (D) Sanger sequencing of patient‐derived cells confirmed the presence of the SCN5A variant and its correction in CRISPR‐edited isogenic controls. (E) Immunofluorescence analysis of pluripotency markers in the BrS and BrS‐corr hiPSCs. (F) Flow cytometry for pluripotency markers in hiPSCs from BrS and BrS‐corr cells. (G) Expression of cardiac‐specific markers alpha‐actinin, cardiac troponin T (cTnT) and ventricular‐specific MLC2V in BrS and non‐BrS (non‐BrS1 and non‐BrS2) and BrS‐corr human‐induced pluripotent stem cell‐cardiomyocytes (hiPSC‐CMs). (H) Western blot analysis of protein expression level of Na_v_1.5 in BrS compared to non‐BrS and BrS‐corr cells. *n* = 3 (number of independent experiments). One‐way analysis of variance (ANOVA) followed with Holm‐Sidak post‐test for multiple comparisons was used for comparing more than two groups in (H). The data are presented in mean ± standard error.

A 49‐year‐old Caucasian patient applied the hospital due to a knee empyema with a febrile constellation. In the recorded ECG, tent‐like ST‐segment elevations were noticed in the presence of fever in the right precordial leads, fitting a type 1 BrS ECG (Figure 1A). Ventricular fibrillation was easily induced with S1S1 370, S1S2 210 ms. A defibrillator was implanted for primary prevention. Sanger‐sequencing revealed a heterozygous missense variant (c.3148G>A/p. Ala1050Thr) in the *SCN5A* gene (Figure 1C). Dermal skin fibroblasts of the patient were successfully reprogrammed into hiPSCs. Further, we generated a site‐corrected iPSC line by CRISPR/Cas9 genome editing (Figure 1B,D). The hiPSCs displayed characteristic human embryonic stem cell morphology and expression of pluripotency markers (Figure 1E,F). The hiPSCs were differentiated into hiPSC‐CMs by WNT signalling modulation. hiPSC‐CMs expressed cardiac‐specific markers including α‐actinin, cTnT and MLC2V (Figure 1G). The mRNA expression of cardiac makers and ion channels in four cell lines (BrS, BrS‐corr, non‐BrS1 and non‐BrS2) showed no significantly different except *SCN5A* expression (Figure S1 A–C).

### 3.2 Possible significance of the *SCN5A* variant (c.3148G>A/p.Ala1050Thr) for pathogenicity

In the ClinVar‐Database, the present variant (c.3148G>A/p. Ala1050Thr) in *SCN5A* was reported in a BrS patient and in another patient with ventricular tachyarrhythmias. The significance was speculated as a variant of unclear significance.^17^, ^18^ According to the ClinGen predictor‐database based on a computer modelled algorithm, the REVEL‐score is 0.73. Of note, threshold scores for use of meta‐predictor tools have not yet been defined. But nevertheless, a REVEL‐score ≥0.7 seems to be consistent with pathogenicity.^19^ On the other hand, according to Cardioboost database, the possibility that the present variant is pathogenic is low, 24.6% (Table 1).

**TABLE 1:** Pathogenicity of the SCN5A variant (c.3148G>A/p.Ala1050Thr)

	ID	Probability of pathogenicity	Related score	Clinical significance	Resources
ClinVar	NM_000335.5	/	/	Uncertain significance	https://www.ncbi.nlm.nih.gov/clinvar
ClinGen pathogenicity calculator	CA352139286	/	/	Undetermined	https://calculator.genome.network/site/cg‐calculator
CardioBoost	/	.24623	/	Uncertain significance	https://www.cardiodb.org/cardioboost/
REVEL‐score	/	/	.73#	Likely pathogenicity	https://doi.org/10.1101/2022.03.17.484479

*Note*: ^#^According to the ClinGen predictor‐database based on a computer modelled algorithm, the REVEL‐score is 0.73. Of note, threshold scores for use of meta‐predictor tools have not yet been defined. But nevertheless, a REVEL‐score ≥ 0.7 seems to be consistent with pathogenicity.

### 3.3 Changes of I_Na_ in hiPSC‐CMs from the BrS patient

**FIGURE 2:**
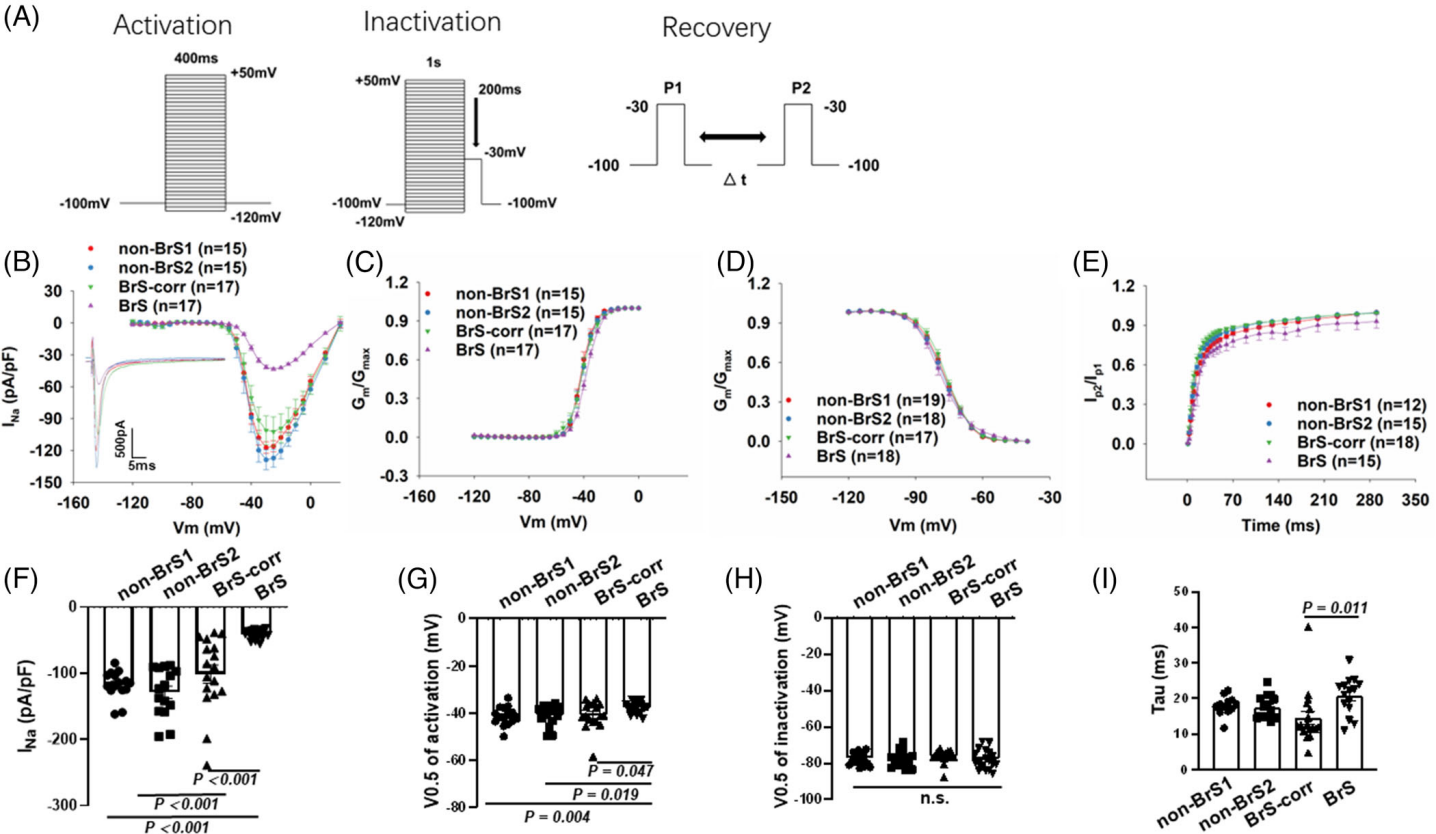
The human‐induced pluripotent stem cell‐cardiomyocytes (hiPSC‐CMs) from BrS patient displayed loss‐of‐function of sodium channels. Peak sodium channel currents were recorded at room temperature in BrS and non‐BrS (non‐BrS1 and non‐BrS2) and BrS‐corr hiPSC‐CMs. (A) Protocols for measuring peak sodium channel currents (I_Na_). (B) Current‐voltage (I‐V) relationship curves of peak I_Na_ in hiPSC‐CMs from each group. (C) Activation curves of peak I_Na_ in hiPSC‐CMs from each group. (D) Inactivation curves of peak I_Na_ in each group. (E) Recovery curves of peak I_Na_ in each group. (F) Mean values of peak I_Na_ at ‐30 mV in hiPSC‐CMs from each group. (G) Mean values of potential at 50% activation (V0.5) in each group. (H) Mean values of potential at 50% inactivation (V0.5) in each group. (I) Mean values of time constant (Tau) of recovery from inactivation in each group. Numbers given in (B–E) represent numbers of measured cells also for (F–I), respectively. One‐way analysis of variance (ANOVA) followed with Holm‐Sidak post‐test for multiple comparisons was used for comparing more than two groups in (F, H, I). The unpaired Student's *t*‐test was used to compare two independent groups in (G). The data are presented in mean ± standard error.

Consistent with the reduction of Na_v_1.5 protein expression levels in cells (Figure 1H), peak sodium current I_Na_ was significantly reduced in BrS cardiomyocytes as compared to non‐BrS and BrS‐corr cells (Figure 2B,F Figure S2A–D). While the activation curves of I_Na_ of BrS were shifted to more positive potentials (Figure 2C,G), the inactivation curves were not significantly shifted (Figure 2D,H). The recovery curves from inactivation of BrS were decelerated (Figure 2E,I). In addition, the membrane protein levels of Nav1.5 in four cell lines (BrS, BrS‐corr, non‐BrS1 and non‐BrS2) were checked by western blot, and the results showed that the protein level in BrS cell membrane was also reduced compared with that in BrS‐corr and non‐BrS cell membrane (Figure S1D,E). These data indicate that the variant in *SCN5A* led to a loss‐of‐function of sodium channels.

### 3.4 Changes of action potential and spontaneous calcium transients in hiPSC‐CMs from the BrS patient

**FIGURE 3:**
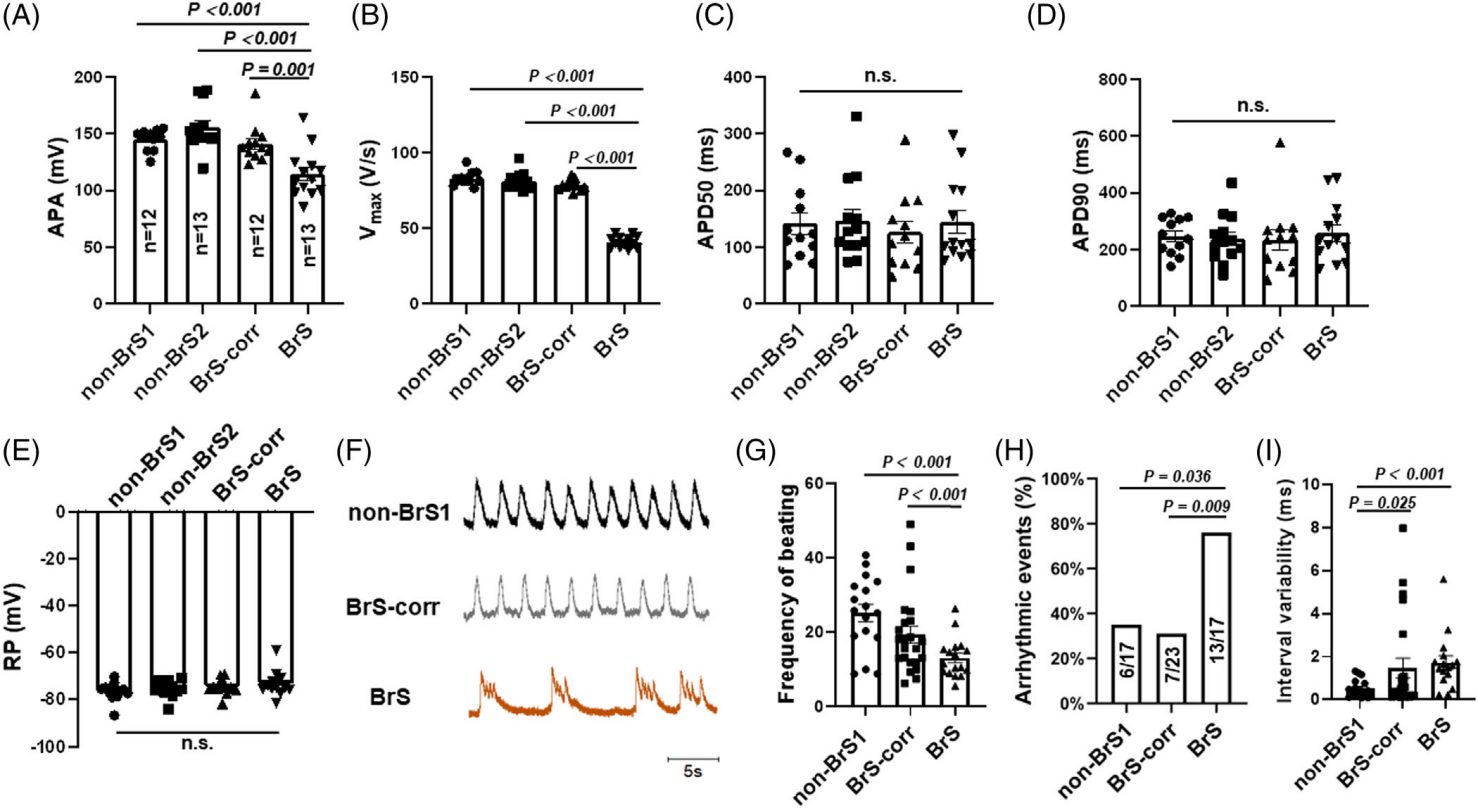
Changes of action potential and calcium transient in human‐induced pluripotent stem cell‐cardiomyocytes (hiPSC‐CMs) from the BrS patient. Action potentials (A–E) and spontaneous calcium transients (F–H) were recorded at room temperature in BrS and non‐BrS (non‐BrS1 and non‐BrS2) and BrS‐corr hiPSC‐CMs. (A) Mean values of the amplitude (APA) of APs in each group. (B) Mean values of maximal depolarization velocity (V_max_) of APs in each group. (C) Mean values of repolarization at 50% (APD50) of APs in hiPSC‐CMs of each group. (D) Mean values of repolarization at 90% (APD90) of APs in hiPSC‐CMs of each group. (E) Mean values of the resting potential (RP) in each group. (F) Traces of spontaneous calcium transients of non‐BrS1, BrS and BrS‐corr cells. (G) The beating frequency in BrS is slower compared to non‐BrS1 and BrS‐corr cells. (H) The presence of arrhythmic events (EAD‐like events or triggered events) is more common in BrS hiPSC‐CMs than non‐BrS1 and BrS‐corr cells. (I) Interval variability in BrS is higher compared to non‐BrS1 and BrS‐corr cells. One‐way analysis of variance (ANOVA) followed with Holm‐Sidak post‐test for multiple comparisons was used for comparing more than two groups in (B–E, I). The unpaired Student's *t*‐test was used to compare two independent groups in (A and G). The Fisher‐test was applied for comparing categorical variables in (H). The data are presented in mean ± standard error.

Consistent with the reduced peak I_Na_, the maximum depolarization velocity (V_max_) and action potential amplitude (APA) were significantly reduced in BrS (Figure 3A–B). The other action potential characteristics including the resting potential (RP), the action potential duration at 50% (APD 50) and 90% (APD 90) repolarization were similar in the measured hiPSC‐CMs from all groups (Figure 3A–E, Figure S3A–D).

Calcium transients in spontaneous beating hiPSC‐CMs were measured at baseline. BrS hiPSC‐CMs presented more arrhythmia‐like events compared to non‐BrS and BrS‐corr cells (Figure 3F,H). Further, slower beating (Figure 3F,G) and higher interval variability in BrS hiPSC‐CMs were detected (Figure 3I).

### 3.5 Effect of temperature variation on the sodium channel current and the role of protein kinase A

**FIGURE 4:**
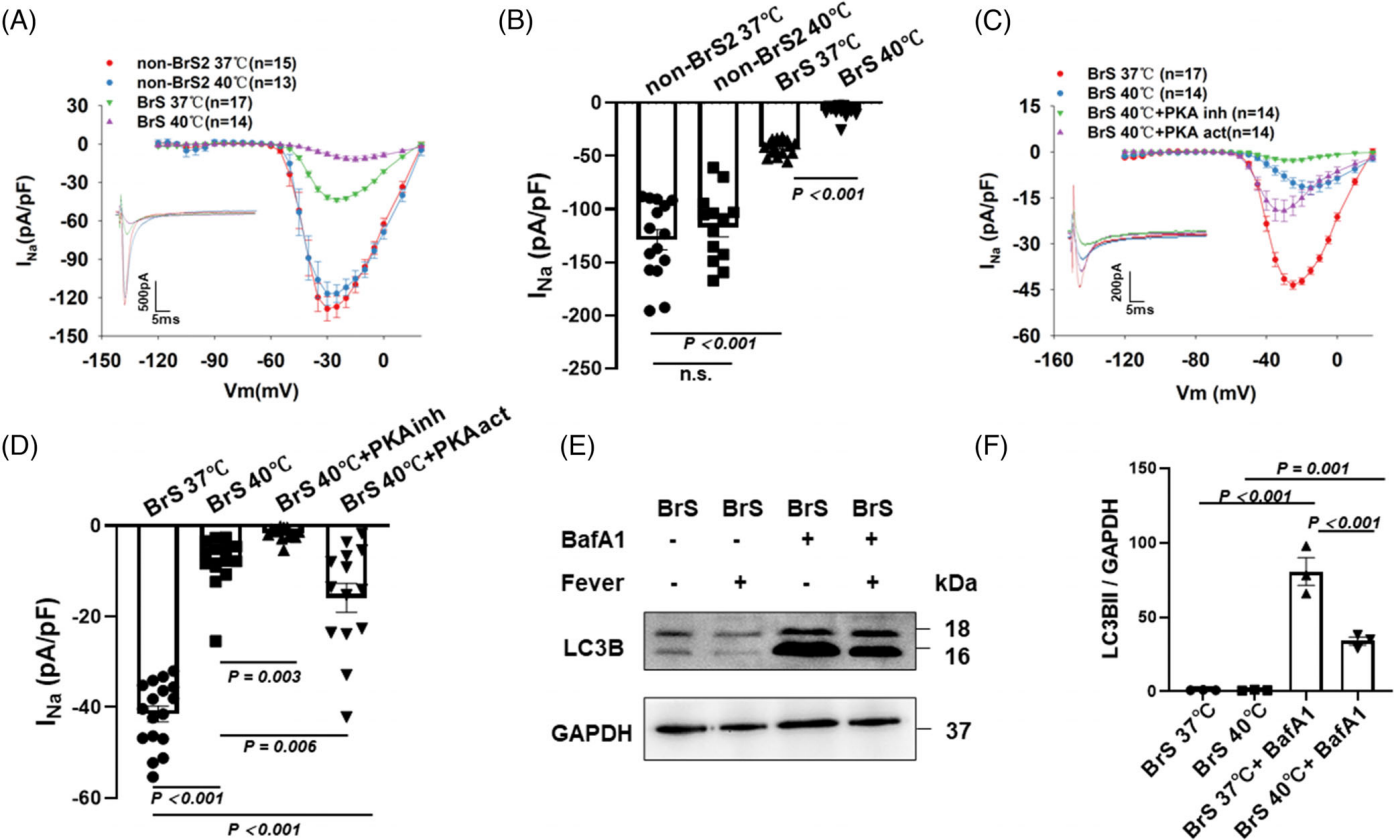
Influence of increasing the temperature of human‐induced pluripotent stem cell‐cardiomyocytes (hiPSC‐CMs) from 37 to 40°C on the I_Na_. Cells were cultured at 37 or 40°C for 24 h and then were used for measurements. (A) IV curves of peak I_Na_ in BrS compared to non‐BrS2 at 37 and 40°C. Representative traces of peak I_Na_ at −30 mV were shown in the inset. (B) Graph bar of BrS peak I_Na_ at −30 mV when temperature is increased from 37 to 40°C showing a significantly reduced sodium channel current in BrS but not in non‐BrS2. (C) IV curves and representative traces at −30 mV (inset) of peak I_Na_ in BrS at 37 and 40°C when the protein kinase A (PKA) activator and the PKA inhibitor were incubated with hiPSC‐CMs for at least 30 min before measurement. (D) Mean values of peak I_Na_ at −30 mV in BrS‐hiPSC‐CMs at 37 and 40°C as well as 40°C with the PKA inhibitor or activator. (E and F) Bands and mean values of wester blots showing autophagy (LC3B II) levels in cells cultured at 37 and 40°C in absence and presence of BafA1 (50nM),*n* = 3 (number of independent experiments). The *n*‐numbers represent numbers of measured cells. The *n*‐numbers in A and C represent also cell numbers for (B) and (D), respectively. One‐way analysis of variance (ANOVA) followed with Holm‐Sidak post‐test for multiple comparisons was used for comparing more than two groups in (B and F). The unpaired Student's *t*‐test was used to compare two independent groups in (D). The data are presented in mean ± standard error.

Since the patient presented type 1 BrS ECG at fever, we tested the effect of temperature variation (37 to 40°C) on the electrical property of BrS hiPSC‐CMs. No acute effect of temperature variation (within 30 min) on the peak current, activation, inactivation and recovery of I_Na_ were detected in BrS cells (Figure S4B–E). Subsequently, we tested the effect of chronic (24 h) temperature variation. Increasing the culture temperature from 37 to 40°C reduced significantly the peak I_Na_ in BrS‐hiPSC‐CMs but not in non‐BrS cells (Figure 4A,B) and suppressed the activation and showed a delayed recovery (Figure S5A,D), decelerated inactivation and showed on effect on recovery (Figure S5B,C,E,F). Consistent with results of peak I_Na_, the expression level of Nav1.5 was further decreased in BrS cell line after increasing the culture temperature but not in non‐BrS and BrS‐corr cell line (Figure S6A,B). In addition, we also checked the temperature effects of 40°C for 48 h and 42°C for 24 h on peak I_Na_ in BrS‐hiPSC‐CMs. However, the results showed no significantly difference in these two groups compared to BrS‐hiPSC‐CMs with 40°C for 24 h (Figure S6C,D). A protein kinase A (PKA) activator (8‐Bromo‐CAMP, 5 μM) partially rescued the peak I_Na_ at 40°C in BrS hiPSC‐CMs (Figure 4C,D), increased the activation and inactivation but showed no effect on recovery (Figure S7A–C). The PKA inhibitor (H‐89, 10 μM) enhanced the recorded temperature (40°C) effects in BrS‐hiPSC‐CMs, showing further reduction of the peak I_Na_ (Figure 4C,D) and an enhancement of inactivation (Figure S7B). At 37°C, the PKA activator and inhibitor showed similar effect on I_Na_ in BrS‐hiPSC‐CMs (Figure S6E,F). The expression levels of PKA were not significantly changed in non‐BrS and BrS‐corr but reduced in BrS hiPSC‐CMs after increasing the temperature to 40°C (Figure S7D,E). The high temperature did not enhance but rather reduced the autophagy level in BrS cells (Figure 4E,F).

### 3.6 Lipopolysaccharides exacerbated phenotypic changes of BrS‐hiPSC‐CMs

**FIGURE 5:**
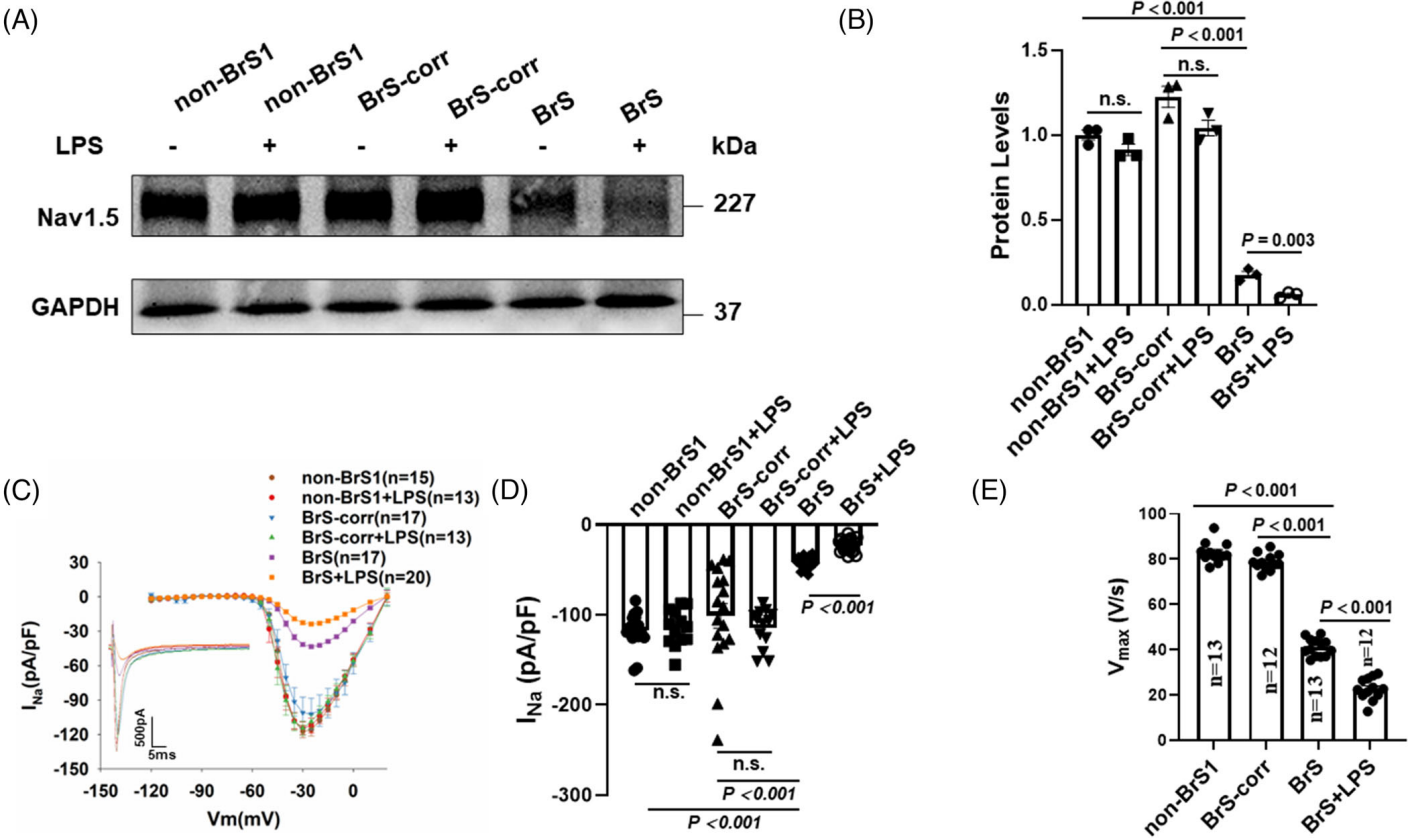
Lipopolysaccharides (LPS) reduced Nav1.5 protein level, peak sodium channel currents and maximal depolarization velocity of action potentials in human‐induced pluripotent stem cell‐cardiomyocytes (hiPSC‐CMs) from the BrS patient. (A and B) Representative (A) and statistical data (B) of western blot showing decreased Nav1.5 expressions after applying LPS (2 μg/ml for 6 h) in BrS cell line, whereas non‐BrS and BrS‐corr cell line showed no significant difference after LPS treatment, *n* = 3 (number of independent experiments). (C) Current‐voltage (I‐V) relationship curves and representative traces at −30 mV (inset) of peak I_Na_ in hiPSC‐CMs of each group. (D) Mean values of peak I_Na_ at −30 mV in hiPSC‐CMs of each group. Numbers given in (D) represent numbers of measured cells for (C) and (D). (E) Mean values of maximal depolarization velocity (V_max_) of APs in hiPSC‐CMs of each group. One‐way analysis of variance (ANOVA) followed with Holm‐Sidak post‐test for multiple comparisons was used for comparing more than two groups in (E). The unpaired Student's *t*‐test was used to compare two independent groups in (B and D). The data are presented in mean ± standard error.

After lipopolysaccharides (LPS) treatment (2 μg/ml, 6 h), the expression level of Nav1.5 was further decreased in BrS cell line but not in non‐BrS and BrS‐corr cell line (Figure 5A,B). I_Na_ was also further reduced after LPS treatment (Figure 5C,D, Figure S2D,E). However, sodium channel gating kinetics were not significantly changed after LPS treatment (Figure S8).

V_max_ was further decreased by LPS in BrS cell line (Figure 5E), while other parameters of APs were not significantly changed (Figure S3D,E, Figure S9A–D).

### 3.7 Reactive oxidative species signalling contributed to LPS effects

**FIGURE 6:**
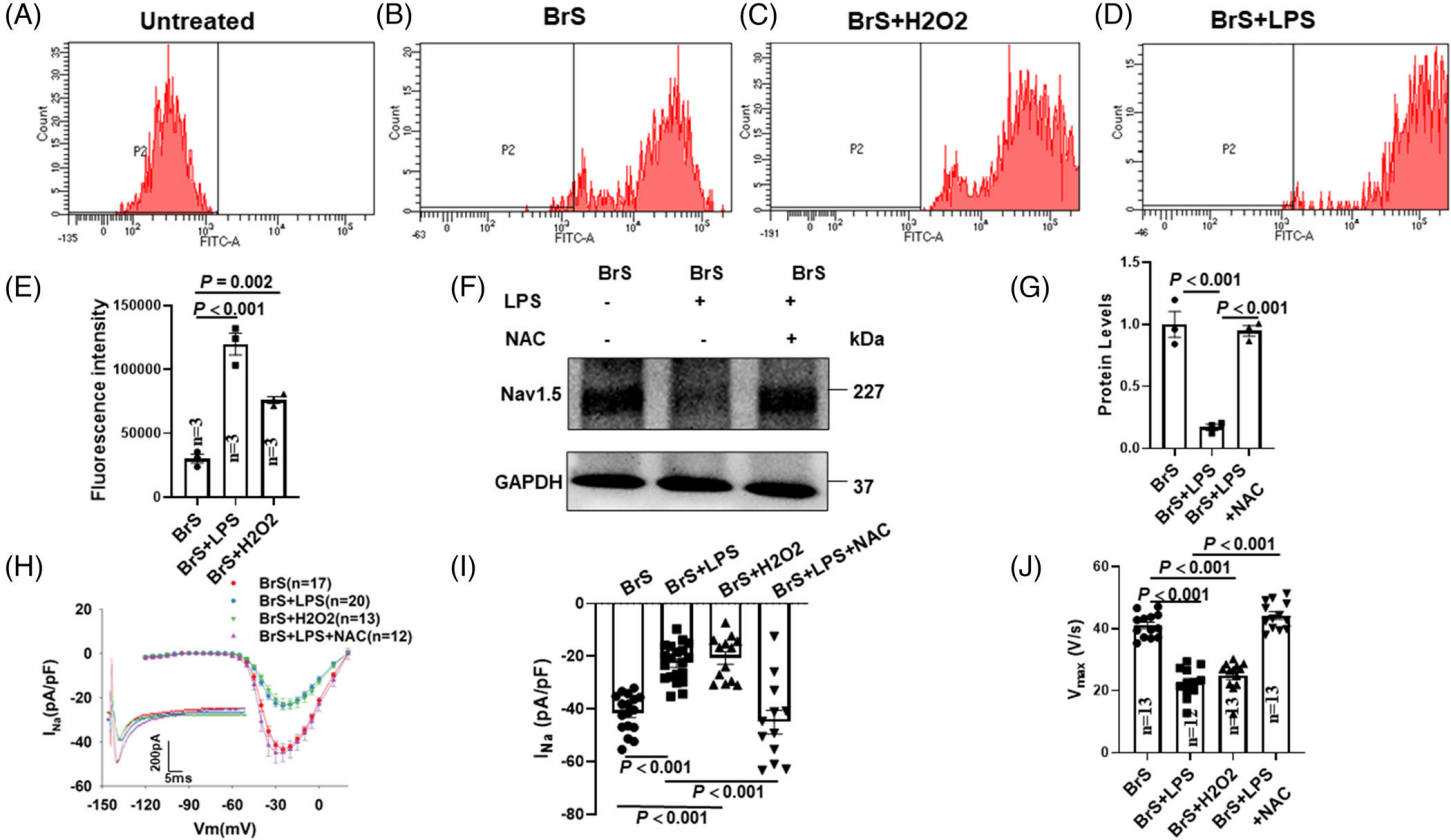
Reactive oxidative species (ROS) is involved in lipopolysaccharides (LPS) effects on Nav1.5 protein level, peak sodium channel currents and action potentials in human‐induced pluripotent stem cell‐cardiomyocytes (hiPSC‐CMs) from the BrS patient. (A) Representative FACS analyses of ROS generation in untreated group. (B) Representative FACS analyses of ROS generation in BrS‐hiPSC‐CMs (control). (C) Representative FACS analyses of ROS generation in BrS‐hiPSC‐CMs with H_2_O_2_ (200μM, 1 h) treatment. (D) Representative FACS analyses of ROS generation in BrS‐hiPSC‐CMs with LPS treatment. (E) Summary of ROS generation from samples in B–D. ‘Untreated’ represents control measurements in cells without treatment of the ROS fluorescence dye, *n* = 3 (number of independent experiments). (F and G) Representative (F) and statistical data (G) of western blot showing decreased Nav1.5 expressions after applying LPS and reversed expression level of Nav1.5 in presence of N‐acetyl‐cystein (NAC) (1 mM) in BrS cell line, *n* = 3 (number of independent experiments). (H) Current‐voltage (I‐V) relationship curves and representative traces at −30 mV (inset) of peak I_Na_ in BrS‐hiPSC‐CMs. (I) Mean values of peak I_Na_ at −30 mV in BrS‐hiPSC‐CMs. Numbers given in (I) represent numbers of measured cells for (H) and (I). (J) Mean values of maximal depolarization velocity (V_max_) of APs in BrS‐hiPSC‐CMs. One‐way analysis of variance (ANOVA) followed with Holm‐Sidak post‐test for multiple comparisons was used for comparing more than two groups in (E, G, I, J). The data are presented in mean ± standard error.

The intracellular reactive oxidative species (ROS) levels were detected by fluorescence activated cell sorting in BrS‐hiPSC‐CMs. After LPS treatment, the ROS production was significantly increased (Figure 6A–E).

An ROS blocker (N‐acetyl‐cystein [NAC], 1 mM) attenuated LPS effects on the expression of Nav1.5 (Figure 6F,G), the peak I_Na_ and V_max_ (Figure 6H–J, Figure S2E,F). H_2_O_2_ (200μM, 1 h), a form of endogenous ROS, simulated LPS effects on I_Na_ and V_max_ (Figure 6H–J, Figure S2D,G). In addition, activation curves of I_Na_ in BrS cells were shifted to more positive potentials by H_2_O_2_ and reversed by NAC (Figure S10A,D). The recovery from inactivation was decelerated in BrS‐hiPSC‐CMs by H_2_O_2_ and reversed by NAC (Figure S10C,F). However, the inactivation curve (Figure S10 B,E) and other parameters of APs (Figure S9E–H) were not significantly changed by H_2_O_2_.

### 3.8 Autophagy was activated by LPS via ROS

**FIGURE 7:**
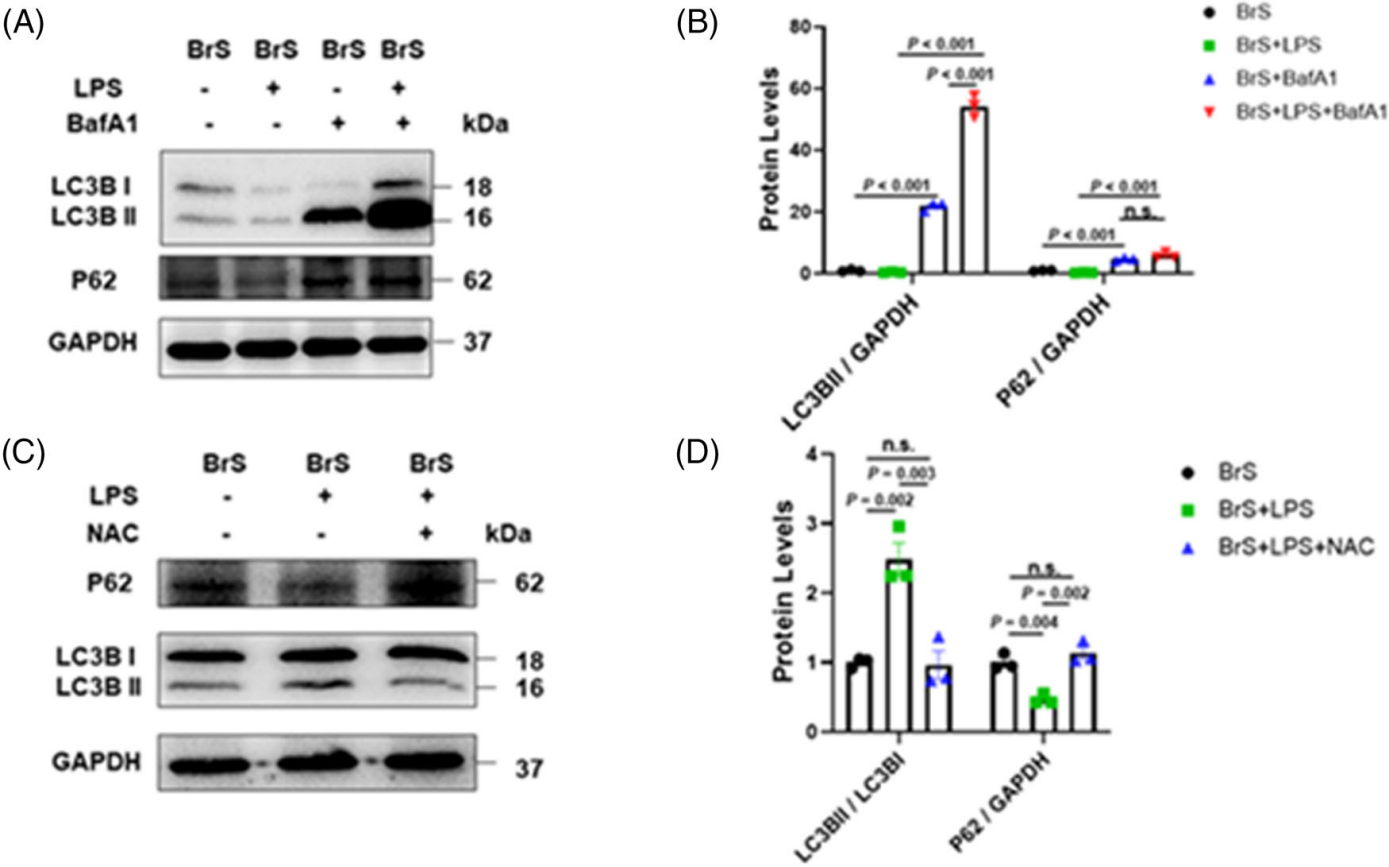
Autophagy was activated after lipopolysaccharides (LPS) treatment by reactive oxidative species (ROS) generation. (A and B) Representative (A) and statistical data (B) of western blot showing increased LC3BII and p62 expressions in cells after treatment with BafA (50 nM), *n* = 3 (number of independent experiments). (C and D) Representative (C) and statistical data (D) of western blot showing increased LC3BII/I ratio and decreased p62 expressions in BrS cell line with LPS treatment and the reversion by N‐acetyl‐cystein (NAC), *n* = 3 (number of independent experiments). One‐way analysis of variance (ANOVA) followed with Holm‐Sidak post‐test for multiple comparisons was used for comparing more than two groups in (B, D). The data are presented in mean ± standard error.

The autophagy flux inhibitor BafA1 (50 nM) was applied to assess the autophagy levels in BrS‐hiPSC‐CMs. In the presence of BafA1, BrS‐hiPSC‐CMs in the absence and presence of LPS both displayed significant increase of LC3BII and p62 compared to that without BafA1, and BrS‐hiPSC‐CMs with LPS plus BafA1 showed higher LC3BII levels than that in BrS‐hiPSC‐CMs only with BafA1 (Figure 7A,B). However, non‐BrS2‐hiPSC‐CMs with LPS plus BafA1 displayed no significant difference of LC3BII levels compared to the non‐BrS2‐hiPSC‐CMs only with BafA1 (Figure S11). These data indicated that LPS could enhance autophagy flux in BrS‐hiPSC‐CMs. In addition, BrS‐hiPSC‐CMs with LPS showed a significant increase of LC3BII/LC3BI ratio and decrease of p62 compared to BrS‐hiPSC‐CMs without LPS, and, interestingly, NAC treatment rescued the effect of LPS on BrS cell line (Figure 7C,D), suggesting the involvement of ROS in the activation of autophagy by LPS.

**FIGURE 8:**
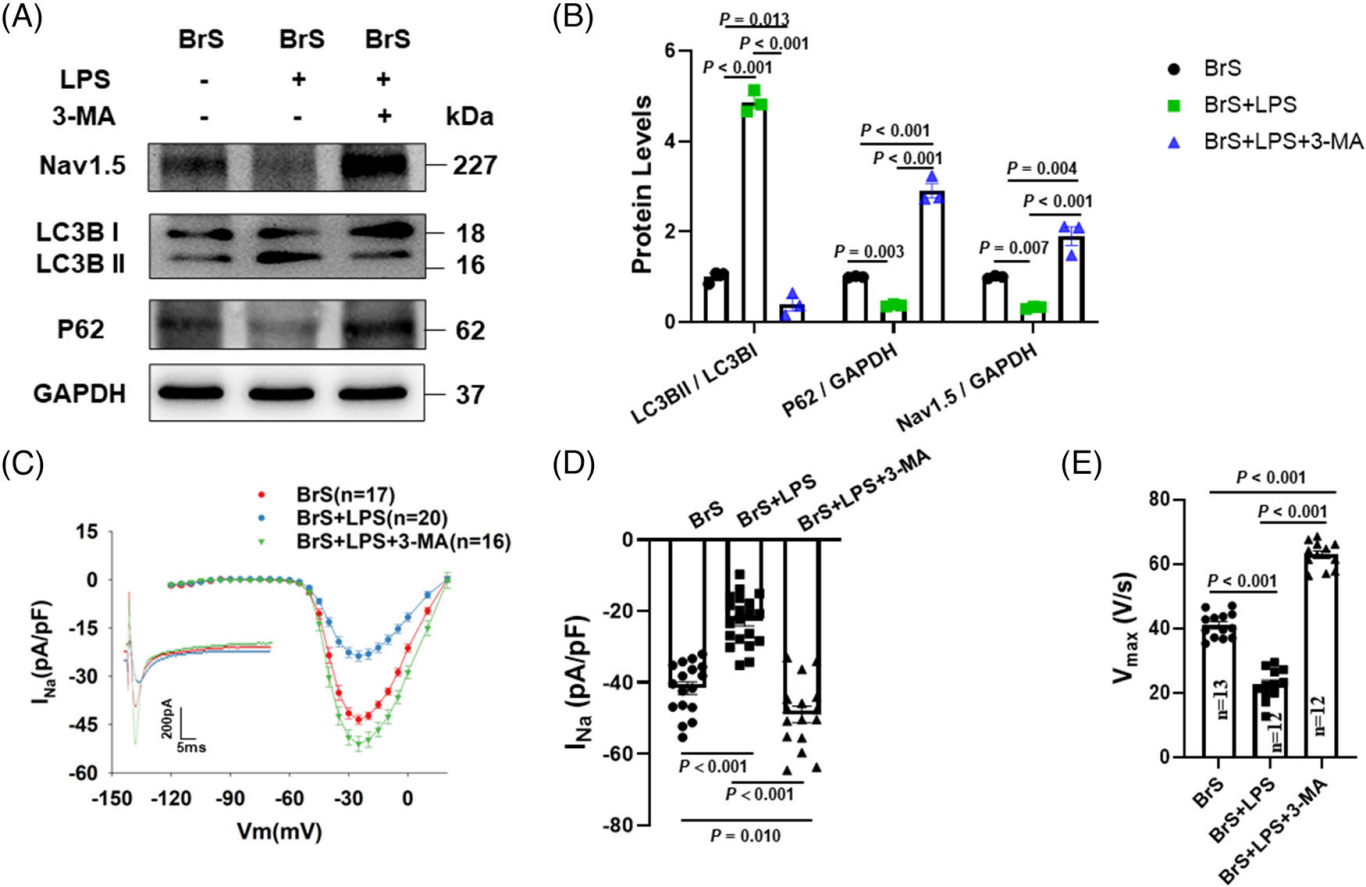
The autophagy inhibitor reversed lipopolysaccharides (LPS) effects on Nav1.5 protein, peak sodium channel currents and action potentials in human‐induced pluripotent stem cell‐cardiomyocytes (hiPSC‐CMs) from the BrS patient. (A and B) Representative (A) and statistical data (B) of western blot showing decreased Nav1.5 expressions, increased LC3BII/I ratio and decreased p62 expressions after applying LPS and the reversion by 3‐MA in BrS cell line, *n* = 3 (number of independent experiments). (C) Current‐voltage (I‐V) relationship curves and representative traces at −30 mV (inset) of peak I_Na_ in BrS‐hiPSC‐CMs. (D) Mean values of peak I_Na_ at −30 mV in BrS‐hiPSC‐CMs. Numbers given in D represent numbers of measured cells for C and D. (E) Mean values of maximal depolarization velocity (V_max_) of APs in BrS‐hiPSC‐CMs. One‐way analysis of variance (ANOVA) followed with Holm‐Sidak post‐test for multiple comparisons was used for comparing more than two groups in (B, D, E). The data are presented in mean ± standard error.

The inhibition of autophagy by 3‐MA (5 mM, 1 h before LPS treatment) could prevent the effect of LPS on Nav1.5 protein level (Figure 8A,B), peak I_Na_ (Figure 8C,D, Figure S2D,E,I) and V_max_ (Figure 8E) in BrS‐hiPSC‐CMs. However, the inhibition of autophagy had no effect on sodium channel kinetic parameters and other AP parameters (Figure S12, Figure S13A–D).

### 3.9 Autophagy was activated by LPS through inhibition of PI3K/Akt/mTOR pathway

**FIGURE 9:**
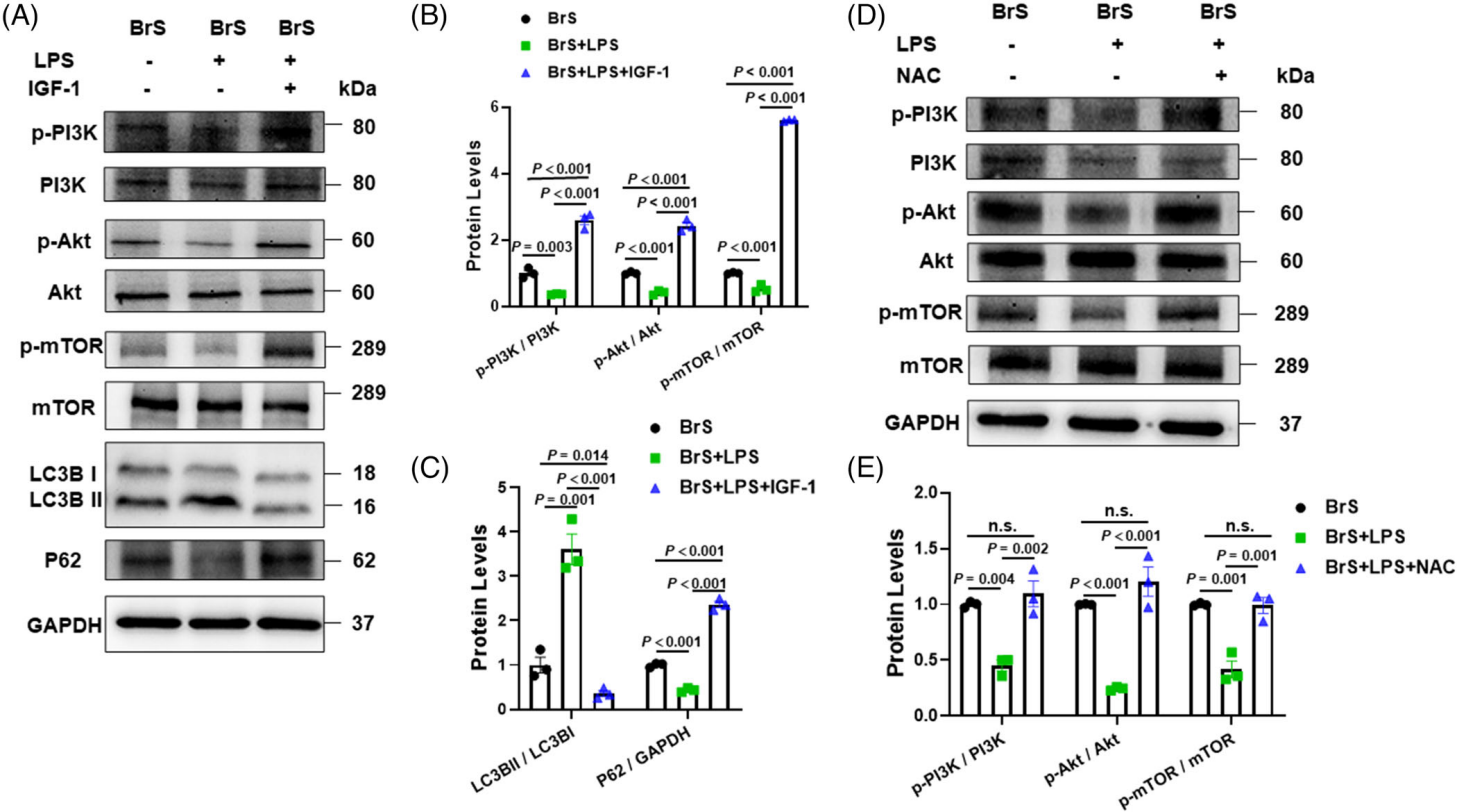
The PI3K/mTOR signalling contributes to the autophagy activation by lipopolysaccharides (LPS) treatment in human‐induced pluripotent stem cell‐cardiomyocytes (hiPSC‐CMs) from the BrS patient. (A and B) Representative (A) and statistical data (B) of western blot showing LPS‐induced decrease and IGF‐1‐induced increase in the phosphorylation of PI3K, Akt and mTOR in hiPSC‐CMs from BrS‐patient. (C) PI3K activator IGF‐1 increased p62 and decreased LC3BII/I ratio expressions in hiPSC‐CMs from BrS‐patients in presence of LPS, *n* = 3 (number of independent experiments). (D and E) Representative (D) and statistical data (E) of western blot showing LPS‐induced decrease and N‐acetyl‐cystein (NAC)‐induced reversion in the phosphorylation of PI3K, Akt and mTOR in hiPSC‐CMs from BrS‐patient, *n* = 3 (number of independent experiments). One‐way analysis of variance (ANOVA) followed with Holm‐Sidak post‐test for multiple comparisons was used for comparing more than two groups in (B, E). The unpaired Student's *t*‐test was used to compare two independent groups in (C). The data are presented in mean ± standard error.

The phosphorylated levels of PI3K, Akt and mTOR were reduced significantly in BrS‐hiPSC‐CMs by LPS (Figure 9A,B). PI3K activator IGF‐1 (100 ng/ml, pre‐treated 2 h before LPS treatment) increased the levels of phosphorylated PI3K, Akt and mTOR and decreased autophagy levels in BrS‐hiPSC‐CMs (Figure 9A–D). NAC treatment prevented the effects of LPS on PI3K, Akt and mTOR in BrS‐hiPSC‐CMs (Figure 9E).

**FIGURE 10:**
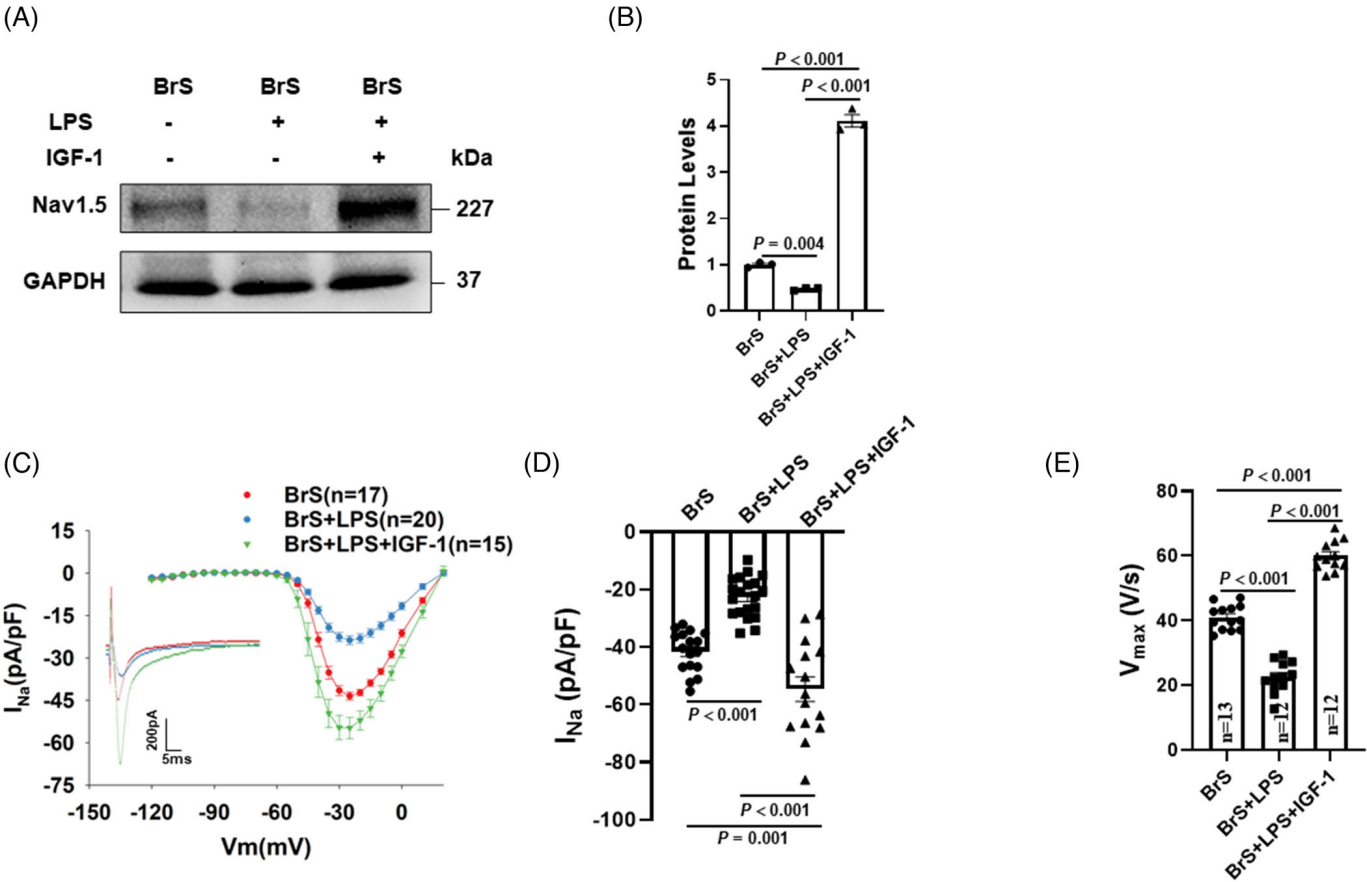
The effect of PI3K/Akt/mTOR signalling activation on peak sodium channel protein and currents and action potentials in human‐induced pluripotent stem cell‐cardiomyocytes (hiPSC‐CMs) from the BrS patient. (A and B) Representative (A) and statistical data (B) of western blot showing decreased Nav1.5 protein expressions after applying lipopolysaccharides (LPS) and the reversion by IGF‐1 in BrS cell line, *n* = 3 (number of independent experiments). (C) Current‐voltage (I‐V) relationship curves and representative traces at −30 mV (inset) of peak I_Na_ in BrS‐hiPSC‐CMs. (D) Mean values of peak I_Na_ at −30 mV in BrS‐hiPSC‐CMs. Numbers given in D represent numbers of measured cells for C and D. (E) Mean values of maximal depolarization velocity (V_max_) of APs in BrS‐hiPSC‐CMs. One‐way analysis of variance (ANOVA) followed with Holm‐Sidak post‐test for multiple comparisons was used for comparing more than two groups in (B, D, E). The data are presented in mean ± standard error.

Nav1.5 protein level was significantly increased by IGF‐1 in BrS‐hiPSC‐CMs in the presence of LPS (Figure 10A,B). The patch‐clamp study showed similar effects in peak I_Na_ (Figure 10C,D, Figure S2 E,J) and V_max_ of APs (Figure 10E). Activation of PI3K/Akt/mTOR pathway had no effect on other AP characteristics (Figure S13E–H) and sodium channel kinetic parameters (Figure S14). In addition, the expression of Nav1.5 protein level and peak I_Na_ were significantly increased in BrS‐hiPSC‐CMs in the presence of IGF‐1 alone (Figure S15A–D), indicating that the activation of PI3K can partially rescue the peak I_Na_ in BrS hiPSC‐CMs. However, IGF‐1 showed no effect on expression of Nav1.5 protein level and peak I_Na_ in non‐BrS and BrS‐corr control cells (Figure S15E–H).

### 3.10 LPS enhanced temperature effect on sodium channel currents

**FIGURE 11:**
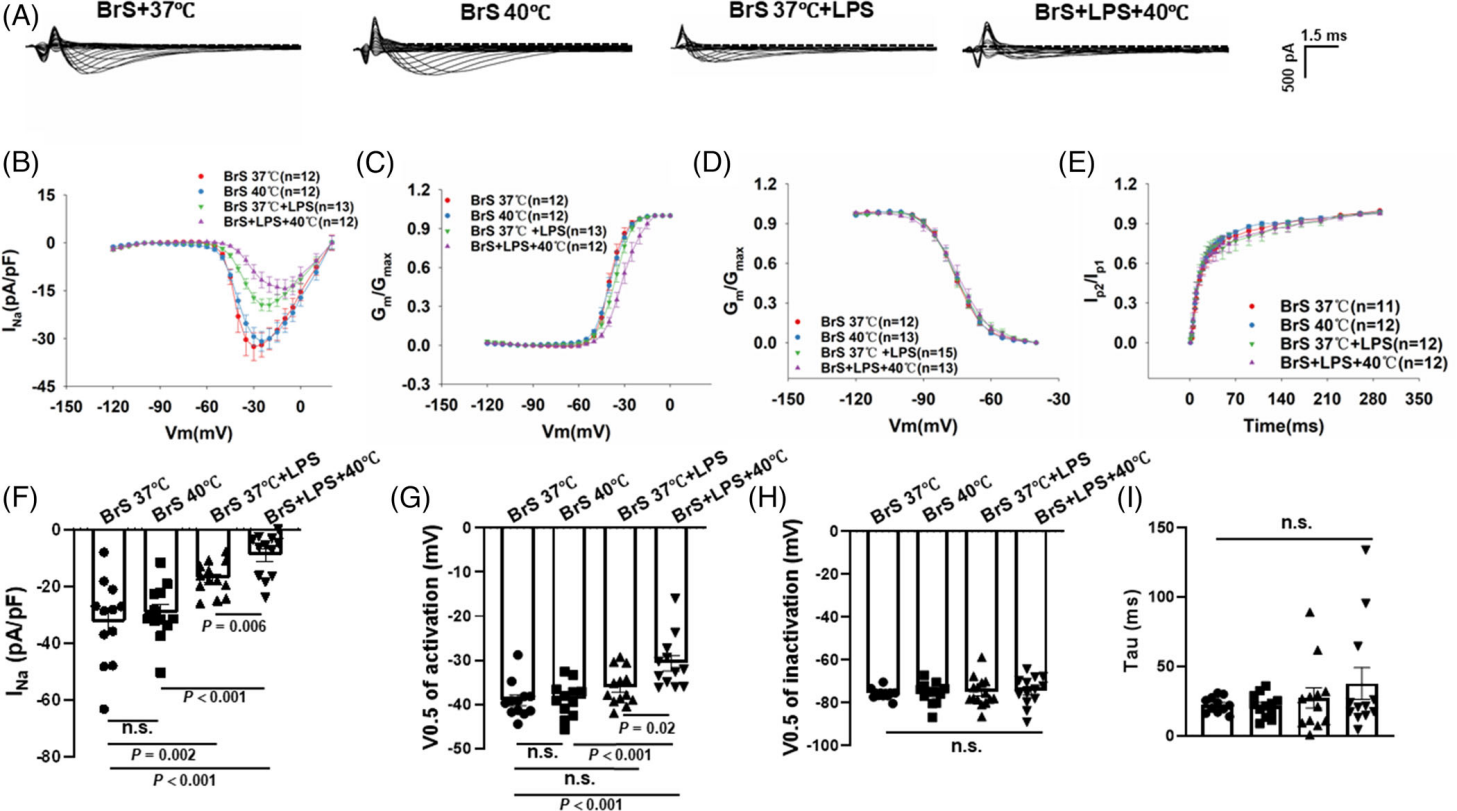
Fever further suppressed peak sodium currents in BrS cells in the presence of lipopolysaccharides (LPS). Peak sodium current (I_Na_) was recorded in BrS‐human‐induced pluripotent stem cell‐cardiomyocytes (hiPSC‐CMs) perfused with solution of 37 (BrS 37°C) and 40°C (BrS + 40°C) in the presence of LPS (BrS 37°C +LPS and BrS+LPS+40°C). (A) Representative traces of peak I_Na_ in BrS cells of each group. (B) Voltage (I‐V) relationship curves of peak I_Na_ in BrS‐hiPS‐CMs of each group. (C) Activation curves of peak I_Na_ in BrS‐hiPS‐CMs of each group. (D) Inactivation curves of peak I_Na_ in BrS‐hiPSC‐CMs from each group. (E) Recovery curves of peak I_Na_ in BrS‐hiPSC‐CMs from each group. (F) Mean values of peak I_Na_ at −30 mV in BrS‐hiPS‐CMs of each group. (G) Mean values of potential at 50% activation (V0.5) in each group. (H) Mean values of potential at 50% inactivation (V0.5) in each group. (I) Mean values of time constant (Tau) of recovery from inactivation in each group. One‐way analysis of variance (ANOVA) followed with Holm‐Sidak post‐test for multiple comparisons was used for comparing more than two groups in (G–I). The unpaired Student's *t*‐test was used to compare two independent groups in (F). The data are presented in mean ± standard error.

Considering that fever is usually accompanied by inflammation, we examined whether LPS could enhance fever effect. Interestingly, when the temperature of perfusion solution to cells pre‐treated with LPS was changed from 37 to 40°C, the peak I_Na_ was further inhibited within 2–3 min (Figure 11). This effect was absent in cells without challenge by LPS (Figure S4), indicating that LPS can enhance the temperature effect on I_Na_.

### 3.11 The effect of hyperthermia and LPS on sodium channel currents of BrS‐hiPSC‐CMs without *SCN5A* mutation

To check whether the effects of hyperthermia and LPS are gene‐variant specific, hiPSC‐CMs from BrS‐patient carrying a variant (c.428C>T/p.Ser143Phec.425C>T/p.S142F) in *CACNB2* (CACNB2‐hiPSC‐CMs) gene, which code a beta‐subunit of L‐type calcium channel, were used for further study. The CACNB2—hiPSC‐CMs showed reduced peak I_Na_ (Figure S16A,B). However, hyperthermia (at 40°C) and LPS failed to reduce peak I_Na_ in this cell line, indicating the hyperthermia and LPS effect is variant‐dependent (Figure S16C,D).

## 4 DISCUSSION

We demonstrate here that the *SCN5A* variant (c.3148G>A/p.Ala1050Thr) can render the channel sensitive to high temperature and LPS challenge and that fever, inflammation and autophagy activation can exacerbate the loss‐of‐function of cardiac sodium channel in BrS with this variant.

In the present paper, we show that the c.3148G>A/p. Ala1050Thr variant is probably pathogenic. The peak sodium current, the protein expression level of Na_v_1.5 and V_max_ were reduced, whereas arrhythmia‐like events were elevated in BrS compared to non‐BrS or BrS‐corr cells, consistent with the cellular and clinical BrS phenotype. Of note, due to the availability limitation, only one patient with this variant was recruited for the study. The real connection between clinical phenotype and this variant needs to be confirmed in future by further studies containing more patient cell lines and more functional assessments.

It has been shown that calcium transient measurements could detect arrhythmia‐like evets in hiPSC‐CMs derived from BrS‐patients.^20^ Indeed, using calcium transient measurements, we could detect arrhythmia‐like events and increased interval variability in BrS‐hiPSC‐CMs, consistent with the previous report. These results together with reduction of peak I_Na_ and V_max_ demonstrated that the hiPSC‐CMs from the patient recapitulated phenotypic features of BrS, and the *SCN5A* variant in the BrS‐patient is probably pathogenic.

Regarding the mechanisms underlying the abnormal calcium transients and arrhythmia‐like events, many questions are still open. The first question is how a mutation/variant in *SCN5A* gene can cause the abnormal calcium handling in BrS‐hiPSC‐CMs. Probably, it is a secondary effect of loss‐of‐function of sodium channels. The loss‐of function of SCN5A channel caused reduction of peak INa, which reduced Vmax of APs. The slower depolarization may result in the reduction of L‐type Ca^2+^ channel activation, which leads to insufficient Ca^2+^ influx, causing smaller calcium release and abnormal calcium handling. In addition, it is known that the high interval variability predisposes to the development of arrhythmia in the tissue or organs. In our BrS‐hiPSC‐CMs, the beating frequency was lower, and interval variability was larger. The lower frequency can prolong action potential/calcium transient duration and hence increase the possibility for occurrence of early afterdepolarizations or early afterdepolarization‐like events. The larger interval variability may increase the possibility for occurrence of extra or irregular beatings. Here, the second question is how the frequency of cell beating was changed by loss‐of function of SCN5A channels in BrS‐hiPSC‐CMs. It was reported that calcium‐activated potassium channels may play important roles for pacemaker activity in cardiac pacemaker cells.^21^, ^22^ Additionally, our recent study demonstrated that SK4 channel (intermediate conductance calcium‐activated potassium channel) is important for the automaticity of hiPSC‐CMs.^23^ The reduced calcium influx caused by slower depolarization in BrS cells may reduce intracellular calcium level and SK4 channel activity and in turn reduce cell automaticity, resulting in slower cell beatings.

Since calcium transients were employed to show a phenotype feature (arrhythmia), and their changes are indirectly related to loss‐of‐function of SCN5A channels, and more importantly, the mechanisms underlying the abnormal calcium transients observed in BrS‐hiPSC‐CMs are unclear, the subsequent experiments with respect to fever and LPS effects on BrS features focused on alterations of SCN5A channels and possible mechanisms for the alterations.

Although the study demonstrated that this variant is very likely pathogenic, further studies using different methods or platforms are needed to confirm its pathogenic role. For example, inserting the variant into a non‐BrS cell line or expressing SCN5A channel of wild type and the variant into a heterologous expression system (HEK or CHO cells) or generating transgenic animals may be helpful to assess the functional consequence of this variant. Computational modelling was demonstrated to be able to model the cardiac electrophysiological activity and ion channel currents including wild‐type (WT) and mutant SCN5A current.^24^, ^25^ The computational modelling may also be useful to assess the pathogenic role of this variant for BrS.^24^

Clinical data showed a type 1 BrS ECG triggered by fever and a higher susceptibility of BrS patients to develop ventricular tachyarrhythmias compared to normal body temperature.^6^ Therefore, current guidelines suggested BrS patients to avoid fever.^26^, ^27^ But nevertheless, the underlying mechanism has not been studied yet. Data in HEK cells in the presence of a BrS mutation overlapped with long‐QT syndrome presented fever‐induced changes of sodium channel current kinetics.^28^ A further study in frog oocytes with the Thr1620Met missense mutant in the *SCN5A* gene presented through changing the temperature from physiological level to 32°C a slowing of recovery from inactivation of I_Na_.^29^ In the present study, we showed that the fever at cellular level exacerbated the BrS phenotype linked to changes of peak I_Na_ and activation kinetic. The reduction of the peak I_Na_ in the presence of a pathogenic variant in *SCN5A* may be linked to mutations in the channel subunits or modifiers of sodium channel function. It has been reported that the GPD1‐L mutation reduced the peak I_Na_ through the activation of protein kinase C (PKC).^30^, ^31^ This suggests that PKC phosphorylation may affect the *SCN5A* and sodium channel current. Other data showed that PKA might impact the reduction of peak I_Na_ in BrS patients.^32^ In the present study, we did not observe an impact of the PKA on the sodium channel current kinetics at physiological temperature. However, in the hiPSC‐CMs at 40°C, the BrS phenotype was exacerbated, for example, lower APA and suppressed activation of peak I_Na_. Importantly, these changes were PKA activity‐dependent. This finding is novel and presents a new insight in the underlying pathomechanism of BrS phenotype at fever. PKA‐inhibiting factors like fever or muscarinic stimulator may reduce cardiac sodium channel currents, triggering BrS phenotype.

It is well‐known that arrhythmias in BrS patients happen frequently at night or rest, suggesting an important role of sympathetic/parasympathetic tonus for the occurrence of arrhythmias. It is also known that the G‐protein‐cAMP‐PKA signalling, as downstream factors of adrenergic and muscarinic receptors, mediate sympathetic and parasympathetic effects on cardiomyocytes. The activation of PKA via neurohumoral stimulation of β‐adrenergic receptors could increase the conduction in normal ventricular myocardium and modulate cardiac electrophysiology,^33^ indicating the possible roles of alterations of PKA and adrenergic stimulation in arrhythmic events.^34^, ^35^ Moreover, catecholamine level in blood can be increased by fever,^36^ suggesting an involvement of activation of adrenoceptor/cAMP/PKA signalling at febrile state. Our data showed that fever reduced I_Na_ in BrS but not healthy hiPSC‐CMs. To understand the reason for the differential effects, we assumed that the cAMP‐PKA signalling may be changed at febrile state or the PKA effect on mutant sodium channels may be altered by fever. Indeed, we detected a hyperthermia‐induced reduction of PKA expression in BrS but not in healthy hiPSC‐CMs. In addition, a family with BrS and an *SCN5A* mutation in a PKA consensus phosphorylation site was identified.^32^ In that study, the in vitro PKA phosphorylation was detected in the I‐II linker peptide of WT channels but not *SCN5A*‐R526H (phosphorylation site) mutant. PKA stimulation significantly increased peak I_Na_ of WT but not mutant channels. Given these findings, we wondered whether the *SCN5A* variant (c.3148G>A/p.Ala1050Thr) in our BrS‐patient could also change the phosphorylation of the channel by PKA. Therefore, we checked PKA effects in BrS‐hiPSC‐CMs. Our results suggested that the *SCN5A* variant ((c.3148G>A/p.Ala1050Thr) in our BrS‐patient probably does not change the phosphorylation of the channel by PKA because the variant channel, like WT channel,^32^ could be activated by PKA activator at both 37 and 40°C. However, the hyperthermia‐induced reduction of PKA expression in BrS but not in healthy cells can help understand the I_Na_ reduction specifically in BrS‐hiPSC‐CMs.

High temperature (fever‐like state) is usually associated with infection. The important roles of inflammation in arrhythmic events have been valued in more and more studies. In patients with acute or chronic myocarditis, inflammatory processes in cardiomyocytes could not only cause fluctuations in membrane potential but also arrhythmias in patients.^37^ In rabbit ischemic heart failure model, anti‐inflammation could increase the levels of Nav1.5 and decrease the inducibility of ventricular arrhythmia.^38^ Inflammation has also been reported to unmask the ECG patterns and exacerbate the malignant ventricular arrhythmias in BrS,^12^, ^14^ but the exact roles and underlying mechanisms of inflammation in the arrhythmogenesis of BrS have not been explored so far. In our study, we used LPS to simulate inflammatory responses in cardiomyocytes. The results showed a significant reduction of the Nav1.5 proteins and peak I_Na_ in BrS cells after exposing to LPS, whereas healthy donor and isogenic cells have not displayed changes, suggesting that inflammation may exacerbate the BrS phenotype.

Of note, the hyperthermia‐ and LPS‐effects were observed only in BrS‐hiPSC‐CMs, suggesting critical role of the gene variants in the diseased cells. To further check whether the effect of hyperthermia and LPS on BrS‐hiPSC‐CMs is variant‐dependent, another BrS cell line without *SCN5A* mutation was utilized. Although this cell line also showed reduced peak I_Na_, hyperthermia, and LPS treatment showed no significant influence on I_Na_ in the BrS cell line without *SCN5A* mutation, indicating the hyperthermia and LPS effect is gene variant‐dependent.

It has been reported that increased ROS production in BrS may induce a lower peak I_Na_.^32^ Accordingly, we detected that LPS challenge increased ROS generation in BrS‐hiPSC‐CMs, ROS blocker abolished and H_2_O_2_ mimicked LPS effects on sodium channel expression or current, indicating an involvement of ROS in LPS effects. However, whether autophagy is a part in the pathomechanism of BrS has not been reported yet. Using western blot analysis, we were able to show that LC3II/I ratio was significantly increased in BrS cells challenged by LPS, indicating that more autophagosomes were generated in the BrS‐hiPSC‐CMs. 3‐MA is one of the commonly used specific autophagy inhibitors, which target PIK3C3 to suppress the formation of PI3KC3 complex. After the treatment with LPS plus 3‐MA, BrS cells displayed a significant increase in Na_v_1.5 expression, suggesting that the LPS‐induced down‐regulation of Na_v_1.5 in BrS‐hiPSC‐CMs was related to autophagy activation. This finding is in agreement with the study of Liu et al.^39^ Their study showed that Na_v_1.5 expression in rat cardiomyocytes was significantly decreased after ischemia and reperfusion injury, which was caused via AMPK‐mediated autophagic activation. Peak I_Na_ and V_max_ of APs were also increased by inhibiting autophagy in BrS cells, suggesting that the excitation propagation in BrS cardiomyocytes can be delayed by activation of autophagy. As the cargo protein for autophagy, p62 will be degraded after autophagy activation. Greatly decreased p62 levels were detected in BrS in the presence of LPS, further confirming the activation of autophagy in BrS by LPS. All these data together suggested that autophagy levels were increased by LPS in cardiomyocytes from BrS. Importantly, NAC prevented the effect of LPS on p62, implying that ROS is involved in LPS‐induced autophagy activation.

Two critical signalling pathways play an important role in regulating autophagy: mTOR‐dependent or ‐independent signalling pathways. mTOR is one of the key regulators of autophagy through affecting the activity of Unc‐51‐like kinase (ULK)1 complex.^40^ The increased phosphorylation level of PI3K/Akt/mTOR signalling pathway can activate mTOR and suppress autophagy via inhibiting the phosphorylation of ULK1.^41^ Recent studies suggested that an inhibition of PI3K/Akt/mTOR signalling could cause a decrease in peak I_Na_ via decreased abundance of Na_v_1.5 on the cell membrane and slower conduction of action potentials.^42^, ^43^ In cardiomyocytes from mice, TGF‐β1 treatment increased peak I_Na_ through enhanced phosphorylation of FOXO1 caused by PI3K/AKT pathway activation.^10^ However, these studies did not explore the relationship among the PI3K/AKT pathway, autophagy and sodium channels. In accordance with these studies, our results displayed that the activation of PI3K/Akt/mTOR pathway by IGF‐1 increased expression of Na_v_1.5, peak I_Na_ and V_max_ of APs in BrS‐hiPSC‐CMs in the presence of LPS. Together, these data suggest that the effect of PI3K/AKT signalling on sodium channels may result from the inhibition of autophagy. LPS enhanced autophagy level in BrS‐hiPSC‐CMs, probably via ROS/PI3K/AKT/mTOR signalling. LPS increased ROS production, which inhibited PI3K/AKT/mTOR signalling and in turn enhanced autophagy level in BrS‐cells, exacerbating the phenotype of BrS.

Although the hyperthermia (24 h) slightly decreased the autophagy levels in BrS cells, the results indicated that it affected sodium channel functions via PKA signalling but not autophagy because decreased autophagy should increase SCN5A expression and I_Na_, which are contrary to the reduced I_Na_ in hyperthermia‐challenged cells, and the hyperthermia‐induced reduction of PKA expression can explain the reduction of peak I_Na_. However, the acute hyperthermia may hardly affect autophagy levels because of the short time. Nevertheless, our results showed that the combination of acute hyperthermia and LPS treatment enhanced the reduction of I_Na_, suggesting a different mechanism, that is, the LPS treatment increased the sensitivity of SCN5A channels to hyperthermia. LPS may change not only sodium channel expression and current but also other physical or chemical features of sodium channels, via different signalling such as ROS and inflammatory factors. It could be possible that some changes of sodium channels, for example, oxidation or phosphorylation, may render the channel more sensitive to hyperthermia. It is known that fever can increase ROS production.^44^ LPS also increased ROS generation, and ROS inhibited sodium channels in BrS‐hiPSC‐CMs. Therefore, on basis of ROS, the effects of fever and LPS could be added when they are combined. This indicates that inflammation may enhance fever effect and may contribute to triggering arrhythmias in BrS. Of note, the effect of mimicked fever (40°C) and LPS on peak I_Na_ was detected in BrS‐ but not non‐BrS cells, suggesting that the variant in *SCN5A* rendered sodium channels sensitive to fever and inflammation challenge. This may help understand the arrhythmogenesis of BrS‐patients with fever or infection.

## 5 CONCLUSIONS

Our results suggest that the variant c.3148G>A/p. Ala1050Thr in *SCN5A* can result in loss‐of‐function of sodium channels and increase the channel sensitivity to fever and inflammation challenge in BrS hiPSC‐CMs. PKA signalling may mediate fever effects, and autophagy can mediate inflammatory effects in BrS cells.

### 5.1 Study limitations

Cell lines from two healthy donors, from one BrS patient, and one CRISPR‐corrected isogenic hiPSC line were used in the main part of study. Possible interindividual variability cannot be excluded. In addition, we cannot rule out the possibility that differences between hiPSC‐CMs and native cardiomyocytes may lead to results different from that in patients. Intrinsic mechanistic factors, hormone rules and epigenetic factors were not taken into consideration regarding regulation of cellular electrophysiology in BrS. Moreover, although LPS is a good in vitro analog for triggering cellular inflammation, it cannot fully mimic the entire circulating inflammatory milieu that exists in vivo. However, these points should be investigated in future studies.

### 5.2 Translational outlook

BrS is a rare heart disease. Patients may present a BrS type 1 electrocardiogram ECG at fever situation and/or inflammation situation. This study demonstrated that the *SCN5A* variant (c.3148G>A/p. Ala1050Thr) caused loss‐of‐function of sodium channels and rendered the channel sensitive to high temperature and inflammation. Our results suggested that inflammation could exacerbate BrS phenotype via enhancing autophagy, whereas fever could exacerbate BrS phenotype via inhibiting PKA‐signalling. This presents new insights in BrS.

## CONFLICT OF INTEREST

The authors declare that there is no conflict of interest that could be perceived as prejudicing the impartiality of the research reported.

## Supporting information

Supporting Information
