# Supplementary material for: Novel insights in the pathomechanism of Brugada syndrome and fever‐related type 1 ECG changes in a preclinical study using human‐induced pluripotent stem cell‐derived cardiomyocytes
Source: Clin Transl Med. 2023 Mar 7;13(3):e1130. doi: 10.1002/ctm2.1130 (PMC9990896; doi:10.1002/ctm2.1130)
Supplement: Supplementary file 1 — Supporting Information [file CTM2-13-e1130-s001.docx]

Supplementary information

# Novel insights in the pathomechanism of Brugada syndrome and fever-related type 1 ECG changes in a preclinical study using human induced pluripotent stem cell-derived cardiomyocytes

**Running title: Pathomechanisms of the SCN5A related BrS changes**

Yingrui Li^1,4^*****, Hendrik Dinkel^1,4^*, Dalia Pakalniskyte^1,4^, Alexandra Viktoria Busley^3,4^, Lukas Cyganek^3,4^, Rujia Zhong^1^, Feng Zhang^1^, Qiang Xu^1,2^, Lasse Maywald^1,4*^, Assem Aweimer^5^, Mengying Huang^1^, Zhenxing Liao^1^, Zhenghui Meng^1^, Chen Yan^1^, Timo Prädel^1,4^, Lena Rose^1^, Alexander Moscu-Gregor^6^, Alyssa Hohn^1^, Zhen Yang^1^, Lin Qiao^1^, Andreas Mügge^5^, Xiaobo Zhou^1,4,2#^ Ibrahim Akin^1,4┼^, and Ibrahim El-Battrawy^1,4┼^

^1^First Department of Medicine, Faculty of Medicine, University Medical Centre Mannheim (UMM), Heidelberg University, 68167 Mannheim, Germany

**^2^Key Laboratory of Medical Electrophysiology of Ministry of Education and Medical Electrophysiological Key Laboratory of Sichuan Province,** Institute of Cardiovascular Research, Southwest Medical University, Luzhou, 646000 Sichuan, China

^3^Stem Cell Unit, Clinic for Cardiology and Pneumology, University Medical Center Göttingen, Göttingen, Germany

^4^DZHK (German Center for Cardiovascular Research), Partner Site, Heidelberg-Mannheim and Göttingen, 68167 Mannheim, Germany

^5^Department of Cardiology and Angiology, Bergmannsheil University Hospitals, Ruhr University of Bochum, Bochum, Germany

^6^Center for Human Genetics and Laboratory Medicine, Martinsried, Germany

*Equally contributed

# Corresponding author.

┼Drs. El-Battrawy and Akin share senior authorship.

Data will be made available on reasonable request.

**Address for correspondence**:

Xiaobo Zhou, MD. First Department of Medicine, University Medical Centre Mannheim, Theodor-Kutzer-Ufer 1-3, 68167 Mannheim, Germany. Phone: 0049-621-383-1448. Fax: 0049-621-383-1474. E-mail: xiaobo.zhou@medma.uni-heidelberg.de

**Materials and Methods**

**Ethics.** The studies generating and using human induced pluripotent stem cells (hiPSCs) and the stem cell derived cardiomyocytes (hiPSC-CMs) were approved by the Medical Ethics Committee II of the University of Heidelberg on 01/05/2010 and 04/12/2012 (2009-350N-MA and 2018-565N-MA). The Ethics Committee of the University Medical Center Göttingen (10/9/15) gave approval on 10/09/2015. The Declaration of Helsinki 1975 of the World Medical Association in its revised version of 2013 served as the basis for the study. Skin biopsies of heart-healthy donors and BrS patients were performed after obtaining written informed consent.

**Genetic study**

Mutations in SCN5A gene were investigated by sequencing of coding exons and conserved parts of splicing regions (+/- 10 bp) through Sanger sequencing, confirmation of results with a second independent approach, data analysis with SeqPilot;
Reference Genome: hg19, NCBI GRCh37; Quality Criteria: SNV detection sensitivity >98%; Classification of Variants: in silico algorithms PolyPhen-2, SIFT, MutationTaster; databases of HGMD Professional release; Limitations: mosaics (<20%).
**Generation of human induced pluripotent stem cells.** HiPSC lines from the BrS patients, from two healthy donors and site-corrected iPSC lines were used in this study. Generation of hiPSC lines was performed at the Stem Cell Unit of the University Medical Center Göttingen. Reprogramming of the BrS patient (with the SCN5A variant) dermal fibroblasts into hiPSC lines isBrSe1.1/10/20 (UMGi127-A clone 1, clone 10, clone 20, abbreviated as BrS) was performed by using the CytoTune-iPS 2.0 Sendai Reprogramming Kit according to established protocols, described previously(1). The hiPSC cell line isBrsd2.1-40 (here abbreviated as BrS-CANCB2) was generated from dermal fibroblasts of a BrS patient with a *CANCB2* variant (c.428C>T/p.Ser143Phe) by using CytoTune-iPS 2.0 Sendai Reprogramming Kit. The non-BrS hiPSC lines ipWT1.1 (UMGi014-B clone 1, here abbreviated as WT1) and isWT11.5 (UMGi130-A clone 5, here abbreviated as WT2) were generated from dermal fibroblasts and peripheral blood mononuclear cells, respectively, using integration-free episomal plasmids or integration-free CytoTune-iPS 2.0 Sendai virus, respectively, and characterized as previously described(2). After verification of pluripotency, the hiPSC lines were frozen and shipped to the laboratory of the First Department of Medicine of the University Medical Centre Mannheim.

**Homology-directed repair (HDR) using CRISPR/Cas9-mediated genome engineering.** Single nucleotide polymorphism c.3148G>A (p. Ala1050Thr) identified in *SCN5A* (Gene ID: 6331) was corrected using CRISPR/Cas9 technology. For performing HDR experiments, undifferentiated hiPSCs from patient cell line isBrSe1.20 (UMGi127-A clone 20) maintained in Essential 8 feeder-free culture medium were transferred and adapted to the DEF-CS 500 culture system (TaKaRa Bio, Cat. # Y30010) according to the manufacturer’s recommendations. For transfection, hiPSCs were seeded at 4x10^6^ cells/well in a 6-well-plate and cultured for 48 h without medium exchange. After two days hiPSCs were collected and seeded at 8x10^6^ cell/well in a 6-well plate and left untouched for 24 h. At day of transfection, medium was exchanged with fresh medium containing 30 µM Alt-R® HDR Enhancer (IDT, Cat. # 1081072). Transfection was performed using Lipofectamine™ CRISPRMAX™ Transfection Reagent (Thermo Fisher Scientific, Cat. CMAX00008) according to manufacturer’s protocol. Briefly, per well 125 µl Opti-MEM™ I Reduced Serum Medium (Thermo Fisher Scientific, Cat. 31985062) were mixed with 2 µl EnGen Cas9 NLS [400pmol] (NEB, Cat. M0646T), 4 µl Alt-R® CRISPR-Cas9 sgRNA [10 µM] (TCTGACTCGGCCACAGTGAT, IDT DNA), and 12.5 µl Cas9 Plus™ Reagent and incubated for 5 min at room temperature to assemble the RNP complex and supplemented with 1.33 µl single stranded Alt-R HDR Donor Oligo (100 µM) (5´CTTCTTCTTGGTCATCTGTGTCTGACTCGGCCACAGCGATGGGCACACACACGGGCTCTGGATCCCCGGGGGTGCCCTGGCCTGGTTGCTCGCCTTCCTCAAACCGTGTTTCCTTGCGGGTGGGAGG-3´, IDT DNA). In the meantime, 25 µl Opti-MEM™ I (Thermo Fisher Scientific, Cat. 31985062) and 7.5 µl CRISPRMAX™ Reagent were mixed, added to the RNA complex, incubated for further 10 min at room temperature and added to the cells. At around 18 h post-transfection, the medium containing the transfection reagent was removed and replaced with fresh medium from Cellartis DEF-CS 500 culturing system. For single-cell cloning, transfected hiPSCs were collected at 96 h post-transfection and plated at 1 cell/well in 96-well plates using Cellartis®iPSC Single-Cell Cloning DEF-CS™ Culture Media Kit (Takara Bio, Cat. Y30021), according to the manufacturer’s recommendations. The cells were incubated as soon as small aggregates of cells were visible. Afterwards individual clonal populations were expanded for further analysis and characterization. For determination of editing, genomic DNA from various clones were isolated using DNeasy Blood and Tissue Kit (Qiagen, Cat.69504) and the SNP containing region was PCR amplified with Q5® High-Fidelity DNA Polymerase (NEB, Cat. M0451S) using following the forward primer (5-AGGGCATGAGTGGTGGATAG-3) and reverse primer (5-CCCCTGATGAGGACAGAGAG-3). For pre-screening, amplicons were first digested using HpyCH4III (NEB, Cat. R0618S) restriction enzyme and successful SNP correction was further confirmed by Sanger-Sequencing (Eurofins). The SNP-corrected hiPSC line isBrSe1-corr.2G6 (UMGi127-A-1 clone 2G6) was verified for pluripotency, as described previously.

**Cell culture system and differentiation of hiPSCs.** The stem cells were thawed and incubated at 37°C in iPS Brew (StemMACS, #130-104-368). After five to seven days, they have been passaged with EDTA into new cell culture dishes. Afterwards, hiPSCs were differentiated into ventricular-like cardiomyocytes according to standardized differentiation protocols(3,4). From day 1 of differentiation, cells received a custom-made serum-free culture medium (RPMI 1640 incl. GlutaMAX + 1% Pen/Strep + 2% B-27 + 0.1% ASC). Targeting the Wnt signaling pathway by GSK3 inhibitor and Wnt activator CHIR99021 (StemMACS #130-103-926) at day 1 and Wnt inhibitor IWP-2/IWP-4 (StemCell #72122/#72552) at day 3 resulted in differentiation into immature cardiomyocytes, which matured in the further course. If cells began beating at day 8 to 10, the differentiation was considered successful. Only cardiomyocytes that were at least at day 45 of differentiation were used for the experiments in this study to ensure adequate gene expression and protein biosynthesis. The differentiation of hiPSCs of each cell line into hiPSC-CMs was performed every two to three weeks and data from different differentiations were combined for statistical analyses. The differentiation of each cell line (non_BrS, BrS and BrS-corr) was parallelly performed using the same protocol under the same condition. The hiPSC-CMs from each cell line with similar differentiation time (45 to 60 days) were used the study. The cell incubation was carried out at 37°C and 40°C for fever experiments. The acute effect of temperature variation was performed by perfusion with solution of 37 ^o^C and 40 ^o^C within 2 minutes. The chronic effect of temperature variation was performed by culturing cells in incubator with 37 ^o^C and 40 ^o^C for 24 hours, as well as 40°C for 48 hours and 42°C for 24 hours. The cell incubation with LPS treatment (2μg/ml, 6 hours) in room temperature was carried out for further experiments.

**Polymerase chain reaction.** For isolation of mRNA and transcription into a double-stranded cDNA, QIAshredder (Qiagen #79656), RNeasy Mini Kit (Qiagen # 74106), DNAse (Qiagen # 79254), p(dT)15-primer (Roche #10814270001) and reverse transcriptase (Roche #10109118001) were used. A microplate reader was utilized for photometric determination of purity and concentration of the mRNA and DNA samples. qPCR was performed by using the specific primers for the genes *SCN5A* (Qiagen # QT00091812), *MYL2* (forward primer- GCAGGCGGAGAGGTTTTC, reverse primer- AGTTGCCAGTCACGTCAGG), cTnT (Qiagen # QT000364), cTnI (forward primer- TTTGACCTTCGAGGCAAGTTT, reverse primer- CCCGGTTTTCCTTCTCGGTG), ACTN2 (forward primer- CAAACCTGACCGGGGAAAAAT, reverse primer- CTGAATAGCAAAGCGAAGGATGA), ACTA2 (Qiagen # QT001103.3), MYH6 (forward primer-GCCCTTTGACATTCGCACTG, reverse primer-GGTTTCAGCAATGACCTTGCC), RYR2 (forward primer-CATCGAACACTCCTCTACGGA, reverse primer-GGACACGCTAACTAAGATGAGGT), SERCA2 (forward primer-CATCAAGCACACTGATCCCGT, reverse primer-CCACTCCCATAGCTTTCCCAG), MYL3 (forward primer-TCACACCTGAGCAGATTGAAGA, reverse primer-GCTGGAGCATAGGCAGGAAAG), NKX2-5(forward primer-ACCTCAACAGCTCCCTGACTC, reverse primer-ATAATCGCCGCCACAAACTCTCC), SCN10A(forward primer-TCCCTCGAAACTAACAACTTCCG, reverse primer-TCTGCTCCCTATGCTTCTCTC), KCNQ1(forward primer-GGAGCCACACTCTGCTGTC, reverse primer-CTTACAGAACTGTCATAGCCGTC), KNCH2(forward primer-GGCTCATGACACCAACCAC, reverse primer-TTCAGGCGGAAGGTCTTG), KCNJ2(forward primer-GTGTCCGAGGTCAACAGCTT, reverse primer-GGTTGTCTGGGTCTCAATGG), KCND3(forward primer-GGAGACATGGTGCCTAAGACG, reverse primer-TGGTAAATCCGGCTAAAGTTGG), CACNA1C(forward primer-AATCGCCTATGGACTCCTCTT, reverse primer-GCGCCTTCACATCAAATCCG) and *GAPDH* (Qiagen #QT00079247) plus the SibirRoxHot Master Mix, ROX 0.1 µM (Bioron GmbH #119405) and a dNTP mix (Thermo Fisher Scientific #18427013). Several biological replicates, defined as the cDNA from different individual differentiations of each hiPSC line, were used for the qPCR experiments to obtain high representation for the cell lines. Each biological replicate was always measured in technical duplicate in each experiment. For normalization, the housekeeping gene GAPDH was used. Ct values were compared using the Ct method(5) for final analysis.

**Western blot.** First, total protein extraction was performed by gentle cell lysis using the RIPA buffer (Sigma #R0278-50ML) including protease inhibitors (Sigma-Aldrich #P8340-1ML). The membrane protein extraction was performed by Membrane Protein Extraction Kit (abcam, #ab65400). Total and membrane protein measurement was carried out for a uniform total protein concentration of 15 mg per pocket. In order to divide the proteins only by their size, SDS-PAGE was performed. The Western blots were accomplished with the Bio-Rad Mini-PROTEAN® electrophoresis set (Bio-Rad Laboratories, Inc. #1658004EDU) according to the manufacturer’s instructions and PVDF membranes were used due to their high stability. For the immunological detection of the transferred proteins, a primary antibody was incubated overnight, the conjugated secondary antibody for one hour the next day. To block the non-specific binding sites (e.g. on the membrane), milk powder was used. Na_v_1.5 (abcam #ab62388), PKA (abcam #ab75991), LC3B (Cell Signaling #3868), SQSTM1/p62 (Cell Signaling #5114), PI3K (Cell Signaling #4257), PI3K phospho (Cell Signaling #4228), Akt (Cell Signaling #9272), Akt phospho (Cell Signaling #4060), mTOR (Cell Signaling #2983), mTOR phospho (Cell Signaling #5536), β-tubulin (abcam #ab6046) and GAPDH (HyTest #5G4) primary antibodies as well as GAR (Sigma Aldrich #A0545) and GAM (Sigma Aldrich #A3682) secondary antibodies were used. Using a detection reagent (Thermo Fisher Scientific #33209), the membranes were subsequently exposed in a photochamber, photographed and further analyzed.

**Immunofluorescence.** To detect specific proteins and their distribution patterns in the cell, the cardiomyocytes were first fixed with formaldehyde 4% for 10 minutes at room temperature and then exposed to a penetration buffer (Sigma Aldrich #T8787) for another 10 minutes to allow the antibodies entering the cell. After blocking all non-specific binding sites with BSA 5% for 30 minutes, incubation with the primary antibodies was performed overnight. The GAR and GAM secondary antibodies labeled with fluorescent dyes (Invitrogen #A11008/#A11004/#A11036/#A32723) bound to the primary antibodies the following day and could be visualized with the immunofluorescence microscope. After photographing randomly selected areas, immunofluorescence density was measured. Detection of the proteins α-actinin (Sigma-Aldrich #A7811), cTNT (abcam #ab8295), MYL2 (Novus #NBP1-30249), and LC3B (Cell Signaling #3868) was performed. Cell nuclei were stained with the fluorescent dye DAPI (Sigma-Aldrich #F6057-20ML).

**Patch Clamp.** Electrophysiological measurements in this study were performed on intact, contracting cardiomyocytes at not less than 50 days of differentiation using the whole-cell configuration. In addition to recording action potentials using the current-clamp technique, the current of voltage-gated sodium channels (I_Na_) was examined using the voltage-clamp technique. All studies were carried out at room temperature, or according to experiment at 37°C or 40°C. Each recorded current was first normalized to the cell membrane capacitance, yielding the current density (pA/pF). In the case of the sodium current recording, the current density at -30 mV represented the expected largest sodium current (peak I_Na_). Since the I_Na_ plays the most important role in the generation of a myocardial action potential (phase 0), its kinetics were analyzed closely. Measuring the I_Na_ activation kinetics, the membrane conductance for sodium (g_Na_) was calculated by the formula $g_{Na}=\frac{I_{Na}}{(E-E_{rev})}$ (I_Na_=peak sodium current; E = applied potential; E_rev_ = reversal potential). When the membrane conductance was plotted versus the applied voltage, a sigmoidal IV curve was obtained. For analyzing the I_Na_ inactivation kinetics, the sodium channel availability was examined at the test potential of -30 mV with pre-pulses from -120 to 20 mV. The individual currents were normalized to the largest current (I_m_/I_max_) and then was plotted against the applied voltage of pre-pulses. Again, a sigmoidal plot, the inactivation curve was obtained. The Boltzmann distribution was used to determine the voltage at which the half of the voltage-dependent sodium channels are open or inactivated (V_0.5_). In the analysis of the I_Na_ recovery time constant, a double-pulse protocol with increasing intervals was used. The recorded and normalized currents were fitted with a 1st order exponential function to obtain the time constant (tau) of sodium channel recovery from inactivation indicating the elapsed time until 63% of the channels can be activated again. The signals were recorded at 10 kHz, amplified with a microelectrode amplifier at 2 kHz low-pass filtered and digitized. Subsequently, the data of channel currents and APs were evaluated, graphically plotted and statistical analysis was performed.

The bath solution for peak sodium current (I_Na_) measurements contained (mmol/L): 20 NaCl, 110 CsCl, 1.8 CaCl_2_, 1 MgCl_2_, 10 HEPES, 10 glucose, 0.001 nifedipine, pH 7.4 (CsOH). Microelectrodes were filled with (mmol/l): 10 NaCl, 135 CsCl, 2 CaCl_2_, 3 MgATP, 2 TEA-Cl, 5 EGTA, 10 HEPES, pH7.2 (CsOH). The action potentials (APs) were recorded in paced cardiomyocytes. The holding current was -1 pA/pF and pulses of 3 ms to 1 nA were delivered at 1 Hz to evoke APs. The AP parameters including RP, V_max_, APA, APD50 and APD90 were analyzed. The junction potential was not corrected. The bath solution (PSS) for AP measurements contained (mmol/l): 130 NaCl, 5.9 KCl, 2.4 CaCl_2_, 1.2 MgCl_2_, 11 glucose, 10 HEPES, pH 7.4 (NaOH).

To investigate the involvement of PKA in the pathophysiology of BrS, cardiomyocytes were incubated for 30 minutes with either the PKA activator 8-CPT-cAMP (Sigma Aldric #C3912) or the PKA inhibitor H-89 (Cell Signaling Technology #9844S) before the electrophysiological measurements.

**Calcium transient imaging.** Cells were loaded with the fluorescent Ca^2+^-indicator Fluo-3 AM. The fluorescence of the cells was measured by using a Cairn Optoscan calcium imaging system (Cairn Research, UK). Fluorescence was excited by 488 nm and emitted at 520 nm as described before(6).

**Flow cytometry**

Intracellular ROS levels were detected by using 2′,7′-Dichlorofluorescin diacetate (DCFH-DA, sigma, #D6883). At 50 to 60 days, cardiomyocytes were washed by PBS for three times and incubated with 0.05% Trypsin-EDTA for 3 minutes at 37°C. Then the cells were collected and centrifuged at 250 × g for 5 min at room temperature. Next the cell pellets were collected and cleansed in PBS for 2 times. Then, the cells were incubated with PBS containing 10 μM DCFH-DA at 37°C for 1 hour in dark. Next the cells were cleansed in PBS for 2 times and suspended in 300μL PBS for flow cytometry analysis on BD FACSCanto™ II (Becton Dickinson, Heidelberg, Germany). The BD FACS Diva software (Version 8.0.1) was applied for further analysis.

**Statistical analysis.** Statistical analysis of the different measurements was performed using InStat and SigmaPlot. First, the mean values as well as the standard error were calculated and the current-voltage curves or current-time curves of the patch-clamp measurements were plotted. The Kolmogorov-Smirnov test was used to examine the normal distribution of data. To test for significance, the unpaired Student's t-test was used to compare two independent groups. One-way analysis of variance (ANOVA) followed with Holm-Sidak post-test for multiple comparisons was used for comparing more than two groups. P values<0.05 were considered statistically significant. The Fisher-test was applied for comparing categorical variables. The number of cells (immunohistochemistry, patch clamp) and biological replicates (qPCR, Western blot) was described by the letter *n*.

**Figure legends**

**Figure S1 The mRNA expression levels of cardiac markers and ion channels and the membrane protein expression levels of Nav1.5 in BrS, non-BrS and BrS-corr cell lines.** (A-B) The fold change of mRNA expression levels of cardiac markers in BrS, non-BrS and BrS-corr cell lines, n=3 (number of independent experiments). (C) The fold change of mRNA expression levels of ion channels in BrS, non-BrS and BrS-corr cell lines, n=3 (number of independent experiments). (D) and (E) Representative (A) and statistical data (B) of western blot showing decreased Nav1.5 membrane protein expression levels in BrS cell line compared to non-BrS and BrS-corr cell lines, n=3 (number of independent experiments). One-way analysis of variance (ANOVA) followed with Holm-Sidak post-test for multiple comparisons was used for comparing more than two groups in (A-C, E). The data are presented in mean ± standard error.

**Figure S2 Representative traces of peak sodium channel current.** Peak sodium channel currents (I_Na_) were recorded at room temperature in hiPSC-CMs from non-BrS (non-BrS1 and non-BrS2), the BrS patient (BrS) and BrS-corr cell line. (A)-(B) Representative traces of I_Na_ in hiPSC-CMs from non-BrS (non-BrS1 and non-BrS2). (C) Representative traces of I_Na_ in hiPSC-CMs from BrS-corr cell line. (D) Representative traces of I_Na_ in hiPSC-CMs from the BrS-patient. (E) Representative traces of I_Na_ in hiPSC-CMs from the BrS patient in presence of LPS (2 µg/ml, 6 h). (F) Representative traces of I_Na_ in hiPSC-CMs from the BrS patient in presence of LPS plus NAC (1 mM). (G) Representative traces of I_Na_ in hiPSC-CMs from the BrS patient in presence of H_2_O_2_ (200 µM). (H) Representative traces of I_Na_ in hiPSC-CMs from non-BrS1 in presence of LPS (2 µg/ml, 6 h). (I) Representative traces of I_Na_ in hiPSC-CMs from the BrS patient in presence of LPS plus 3-MA (5 mM). (J) Representative traces of I_Na_ in hiPSC-CMs from the BrS patient in presence of LPS plus IGF-1 (100 ng/ml). (K) Representative traces of I_Na_ in hiPSC-CMs from BrS-corr in presence of LPS (2 µg/ml, 6 h). (L) Representative traces of I_Na_ in hiPSC-CMs from non-BrS2 in presence of 40℃. (M) Representative traces of I_Na_ in hiPSC-CMs from the BrS patient in presence of 40℃. (N) Representative traces of I_Na_ in hiPSC-CMs from the BrS patient in presence of 40℃ plus PKA inhibitor (H-89, 10 µM). (O) Representative traces of I_Na_ in hiPSC-CMs from the BrS patient in presence of 40℃ plus PKA activator (8-Bromo-CAMP, 5 µM).

**Figure S3 Representative traces of action potentials.** Action potentials were recorded with patch clamp whole cell configuration at room temperature in hiPSC-CMs from non-BrS (non-BrS1 and non-BrS2), the BrS patient (BrS) and BrS-corr cell line. (A)-(B) Representative traces of action potentials in hiPSC-CMs from non-BrS (non-BrS1 and non-BrS2). (C) Representative trace of action potential in hiPSC-CMs from BrS-corr cell line. (D) Representative trace of action potential in hiPSC-CMs from the BrS-patient. (E) Representative trace of action potential in hiPSC-CMs from the BrS patient in presence of LPS (2 µg/ml, 6 h). (F) Representative trace of action potential in hiPSC-CMs from the BrS patient in presence of LPS plus NAC (1 mM). (G) Representative trace of action potential in hiPSC-CMs from the BrS patient in presence of H_2_O_2_ (200 µM). (H) Representative trace of action potential in hiPSC-CMs in hiPSC-CMs from the BrS patient in presence of LPS plus 3-MA (5 mM). (I) Representative trace of action potential in hiPSC-CMs from the BrS patient in presence of LPS plus IGF-1.

**Figure S4.** **Acute effects of high temperature on sodium channel currents in hiPSC-CMs from the BrS patient.** Peak sodium channel currents (I_Na_) were recorded in hiPSC-CMs from the BrS patient at 37^o^C first and then change the perfusion solution temperature to 40^o^C and I_Na_ was recorded again within 2 minutes. (A) Mean values of peak I_Na_ at -30 mV in BrS-hiPSC-CMs. (B) Mean values of potentials at 50% activation (V0.5) of activation curves of peak I_Na_ in BrS-hiPSC-CMs. (C) Mean values of potentials at 50% inactivation (V0.5) of inactivation curves of peak I_Na_ in BrS-hiPSC-CMs. (D) Mean values of time constants (Tau) of recovery from inactivation of peak I_Na_ in BrS-hiPSC-CMs. The unpaired Student's t-test was used to compare two independent groups in (A-D). The data are presented in mean ± standard error.

**Figure S5.** **Effects of high temperature on sodium channel currents in hiPSC-CMs from the BrS patient.** Peak sodium channel currents (I_Na_) were recorded in non-BrS (non-BrS1 and non-BrS2) hiPSC-CMs and BrS-hiPSC-CMs cultured for 24 h at 37^o^C or 40^o^C. (A) Activation curves of peak I_Na_ in hiPSC-CMs of each group. (B) Inactivation curves of peak I_Na_ in hiPSC-CMs of each group. (C) Curves of recovery from inactivation of peak I_Na_ in hiPSC-CMs of each group. (D) Mean values of potentials at 50% activation (V0.5) of activation curves of peak I_Na_ in hiPSC-CMs of each group. (E) Mean values of potentials at 50% inactivation (V0.5) of inactivation curves of peak I_Na_ in hiPSC-CMs of each group. (F) Mean values of time constants (Tau) of recovery from inactivation of peak I_Na_ in hiPSC-CMs of each group. One-way analysis of variance (ANOVA) followed with Holm-Sidak post-test for multiple comparisons was used for comparing more than two groups in (D-F). The data are presented in mean ± standard error.

**Figure S6. Effect of high temperature on sodium channels in hiPSC-CMs.** (A) and (B) Representative (A) and statistical data (B) of western blot showing decreased Nav1.5 protein expressions after high temperature treatment in BrS cell line, but not in non-BrS and BrS-corr cell lines, n=3 (number of independent experiments). Peak sodium channel currents (I_Na_) were recorded in BrS-hiPSC-CMs cultured for 24 h at 37^o^C, 40^o^C and 42^o^C, or 48h at 40^o^C. (C) Current-voltage (I-V) relationship curves of peak I_Na_ in hiPSC-CMs of each group. (D) Mean values of peak I_Na_ at -30 mV in hiPSC-CMs of each group. (E) Current-voltage (I-V) relationship curves of peak I_Na_ in BrS-hiPSC-CMs at 37 °C in absence (BrS 37°C) and presence of PKA inhibitor (BrS 37°C+PKA inh) or activator (BrS 37°C+PKA act). (F) Mean values of peak I_Na_ at -30 mV in BrS-hiPSC-CMs of each group. One-way analysis of variance (ANOVA) followed with Holm-Sidak post-test for multiple comparisons was used for comparing more than two groups in (D, F). The unpaired Student's t-test was used to compare two independent groups in (B). The data are presented in mean ± standard error.

**Figure S7.** **Protein kinase A is involved in temperature effect on sodium channels in hiPSC-CMs from the BrS patient.** (A) Mean values of potentials at 50% activation (V0.5) of activation curves of peak I_Na_ in BrS-hiPSC-CMs cultured for 24 h at 37^o^C or 40^o^C (BrS 37^o^C, BrS 40^o^C) or presence of protein kinas inhibitor (PKA inh.) or PKA activator (PKA act). (B) Mean values of potentials at 50% inactivation (V0.5) of inactivation curves of peak I_Na_ in hiPSC-CMs of each group. (C) Mean values of time constants (Tau) of recovery from inactivation of peak I_Na_ in hiPSC-CMs of each group. (D)-(E) Bands and mean values of western blot analyses in hiPSC-CMs from non-BrS (non-BrS1 and non-BrS2), BrS-corr and BrS-patient, which were cultured at 37^o^C and 40^o^C. One-way analysis of variance (ANOVA) followed with Holm-Sidak post-test for multiple comparisons was used for comparing more than two groups in (A-C, E). The data are presented in mean ± standard error.

**Figure S8. LPS showed no effect on sodium channel gating kinetics in hiPSC-CMs.** Peak sodium channel currents (I_Na_) were recorded at room temperature in hiPSC-CMs from non-BrS (non-BrS1 and non-BrS2), the BrS patient (BrS) and BrS-corr cell line in absence or presence of LPS. (A) Activation curves of peak I_Na_ in hiPSC-CMs of each group. (B) Inactivation curves of peak I_Na_ in hiPSC-CMs of each group. (C) Recovery curves of peak I_Na_ in hiPSC-CMs of each group. (D) Mean values of potential at 50% activation (V0.5) in hiPSC-CMs of each group. (E) Mean values of potential at 50% inactivation (V0.5) in hiPSC-CMs of each group. (F) Mean values of time constant (Tau) of recovery from inactivation in hiPSC-CMs of each group. Numbers given in D-F represent numbers of measured cells also for A-C. The unpaired Student's t-test was used to compare two independent groups in (D-F). The data are presented in mean ± standard error.

**Figure S9. Effects of LPS, ROS and ROS blocker on action potential parameters in hiPSC-CMs.** Action potentials were recorded with patch clamp whole cell configuration at room temperature in hiPSC-CMs from the BrS patient (BrS) in absence or presence of LPS, H_2_O_2_ or ROS blocker NAC. Values in hiPSC-CMs from non-BrS (non-BrS1) and BrS-corr cell line were shown as control. (A) and (E) Mean values of amplitude of APs (APA) in hiPSC-CMs of each group. (B) and (F) Mean values of the resting potential (RP) in hiPSC-CMs of each group. (C) and (G) Mean values of repolarization at 50% (APD50) of APs in hiPSC-CMs of each group. (D) and (H) Mean values of repolarization at 90% (APD90) of APs in hiPSC-CMs of each group**.** One-way analysis of variance (ANOVA) followed with Holm-Sidak post-test for multiple comparisons was used for comparing more than two groups in (A-H). The data are presented in mean ± standard error.

**Figure S10. Effects of LPS, ROS and ROS blocker on sodium channel gating kinetics in BrS-hiPSC-CMs.** Peak sodium channel currents (I_Na_) were recorded with patch clamp whole cell configuration at room temperature in hiPSC-CMs from the BrS patient (BrS) in absence or presence of LPS, H_2_O_2_ or ROS blocker NAC. (A) Activation curves of peak I_Na_ in BrS-hiPSC-CMs of each group. (B) Inactivation curves of peak I_Na_ in BrS-hiPSC-CMs of each group. (C) Recovery curves of peak I_Na_ in BrS-hiPSC-CMs of each group. (D) Mean values of potential at 50% activation (V0.5) in BrS-hiPSC-CMs of each group. (E) Mean values of potential at 50% inactivation (V0.5) in BrS-hiPSC-CMs of each group. (F) Mean values of time constant (Tau) of recovery from inactivation in BrS-hiPSC-CMs of each group. Numbers given in D-F represent numbers of measured cells also for A-C. One-way analysis of variance (ANOVA) followed with Holm-Sidak post-test for multiple comparisons was used for comparing more than two groups in (D-F). The data are presented in mean ± standard error.

**Figure S11. Effects of LPS on autophagy flux in non-BrS-hiPSC-CMs.** (A) and (B) Representative (A) and statistical data (B) of western blot showing no significant difference of LC3BII levels in non-BrS-hiPSC-CMs after treatment with LPS (2 μg/ml for 6 h) plus BafA1 (50 nM) compared to the cells only with BafA1, n=4 (number of independent experiments). One-way analysis of variance (ANOVA) followed with Holm-Sidak post-test for multiple comparisons was used for comparing more than two groups in (B, D). The data are presented in mean ± standard error.

**Figure S12. Effects of LPS and autophagy inhibitor on sodium channel gating kinetics in BrS-hiPSC-CMs.** Peak sodium channel currents (I_Na_) were recorded with patch clamp whole cell configuration at room temperature in hiPSC-CMs from the BrS patient (BrS) in absence or presence of LPS and LPS plus the autophagy inhibitor 3-MA. (A) Activation curves of peak I_Na_ in BrS-hiPSC-CMs of each group. (B) Inactivation curves of peak I_Na_ in BrS-hiPSC-CMs of each group. (C) Recovery curves of peak I_Na_ in BrS-hiPSC-CMs of each group. (D) Mean values of potential at 50% activation (V0.5) in BrS-hiPSC-CMs of each group. (E) Mean values of potential at 50% inactivation (V0.5) in BrS-hiPSC-CMs of each group. (F) Mean values of time constant (Tau) of recovery from inactivation in BrS-hiPSC-CMs of each group. Numbers given in D-F represent numbers of measured cells also for A-C. One-way analysis of variance (ANOVA) followed with Holm-Sidak post-test for multiple comparisons was used for comparing more than two groups in (D-F). The data are presented in mean ± standard error.

**Figure S13. Effects of LPS, 3-MA and IGF-1 on action potential parameters in BrS-hiPSC-CMs.** Action potentials were recorded with patch clamp whole cell configuration at room temperature in hiPSC-CMs from the BrS patient in absence (BrS) or presence of LPS (BrS+LPS), or LPS plus 3-MA (BrS+LPS+3-MA) or LPS plus IGF-1 (BrS+LPS+IGF-1). (A) and (E) Mean values of amplitude of APs (APA) in hiPSC-CMs of each group. (B) and (F) Mean values of the resting potential (RP) in hiPSC-CMs of each group. (C) and (G) Mean values of repolarization at 50% (APD50) of APs in hiPSC-CMs of each group. (D) and (H) Mean values of repolarization at 90% (APD90) of APs in hiPSC-CMs of each group**.** One-way analysis of variance (ANOVA) followed with Holm-Sidak post-test for multiple comparisons was used for comparing more than two groups in (A-H). The data are presented in mean ± standard error.

**Figure S14. Effects of LPS and IGF-1 on sodium channel gating kinetics in BrS-hiPSC-CMs.** Peak sodium channel currents (I_Na_) were recorded with patch clamp whole cell configuration at room temperature in hiPSC-CMs from the BrS patient in absence (BrS) or presence of LPS (BrS+LPS) or LPS plus IGF-1 (BrS+LPS+IGF-1). (A) Activation curves of peak I_Na_ in BrS-hiPSC-CMs of each group. (B) Inactivation curves of peak I_Na_ in BrS-hiPSC-CMs of each group. (C) Recovery curves of peak I_Na_ in BrS-hiPSC-CMs of each group. (D) Mean values of potential at 50% activation (V0.5) in BrS-hiPSC-CMs of each group. (E) Mean values of potential at 50% inactivation (V0.5) in BrS-hiPSC-CMs of each group. (F) Mean values of time constant (Tau) of recovery from inactivation in BrS-hiPSC-CMs of each group. Numbers given in D-F represent numbers of measured cells also for A-C. One-way analysis of variance (ANOVA) followed with Holm-Sidak post-test for multiple comparisons was used for comparing more than two groups in (D-F). The data are presented in mean ± standard error.

**Figure S15. Effect of IGF-1 on Nav1.5 protein level and peak sodium channel currents in hiPSC-CMs.** (A) and (B) Representative (A) and statistical data (B) of western blot showing decreased Nav1.5 expressions in BrS cell line and the reversion by IGF-1, n=3 (number of independent experiments). (C) Current-voltage (I-V) relationship curves of peak I_Na_ in hiPSC-CMs of non-BrS and BrS cell line and BrS cells treated with IGF-1. (D) Mean values of peak I_Na_ at -30 mV in hiPSC-CMs of each group. (E) and (F) Representative (E) and statistical data (F) of western blot showing no effect on Nav1.5 expressions in non-BrS and BrS-corr cell lines after IGF-1 treatment, n=4 (number of independent experiments). (G) Current-voltage (I-V) relationship curves of peak I_Na_ in hiPSC-CMs of non-BrS and BrS-corr cell lines in absence and presence of IGF-1. (H) Mean values of peak I_Na_ at -30 mV in hiPSC-CMs of each group. Numbers given in C and G represent numbers of measured cells for D and H. One-way analysis of variance (ANOVA) followed with Holm-Sidak post-test for multiple comparisons was used for comparing more than two groups in (B, F, D, H). The data are presented in mean ± standard error.

**Figure S16. Hyperthermia and lipopolysaccharides displayed no effect on sodium channel currents of BrS-hiPSC-CMs with *CACNB2* variant (c.428C>T/p.Ser143Phe).** Peak sodium (I_Na_) channel currents were recorded at 37°C or 40°C for 24 h or after LPS treatment for 24 h in hiPSC-CMs from the BrS-patient carrying the CACNB2 variant (BrS-CANB2). The non-BrS2 and BrS-CACNB2-corr hiPSC-CMs were used for comparison. (A, C) Current-voltage (I-V) relationship curves of peak I_Na_ in hiPSC-CMs from each group. (B, D) Mean values of peak I_Na_ at -30 mV in hiPSC-CMs of each group. One-way analysis of variance (ANOVA) followed with Holm-Sidak post-test for multiple comparisons (B, D). The data are presented in mean ± standard error.

**References**

1. Hanses U, Kleinsorge M, Roos L et al. Intronic CRISPR Repair in a Preclinical Model of Noonan Syndrome-Associated Cardiomyopathy. Circulation 2020;142:1059-1076.

2. El-Battrawy I, Lan H, Cyganek L et al. Modeling Short QT Syndrome Using Human-Induced Pluripotent Stem Cell-Derived Cardiomyocytes. J Am Heart Assoc 2018;7.

3. Tiburcy M, Hudson JE, Balfanz P et al. Defined Engineered Human Myocardium With Advanced Maturation for Applications in Heart Failure Modeling and Repair. Circulation 2017;135:1832-1847.

4. Kleinsorge M, Cyganek L. Subtype-Directed Differentiation of Human iPSCs into Atrial and Ventricular Cardiomyocytes. STAR Protoc 2020;1:100026.

5. Schmittgen TD, Livak KJ. Analyzing real-time PCR data by the comparative C(T) method. Nat Protoc 2008;3:1101-8.

6. Trafford AW, Diaz ME, Eisner DA. A novel, rapid and reversible method to measure Ca buffering and time-course of total sarcoplasmic reticulum Ca content in cardiac ventricular myocytes. Pflugers Arch 1999;437:501-3.

**
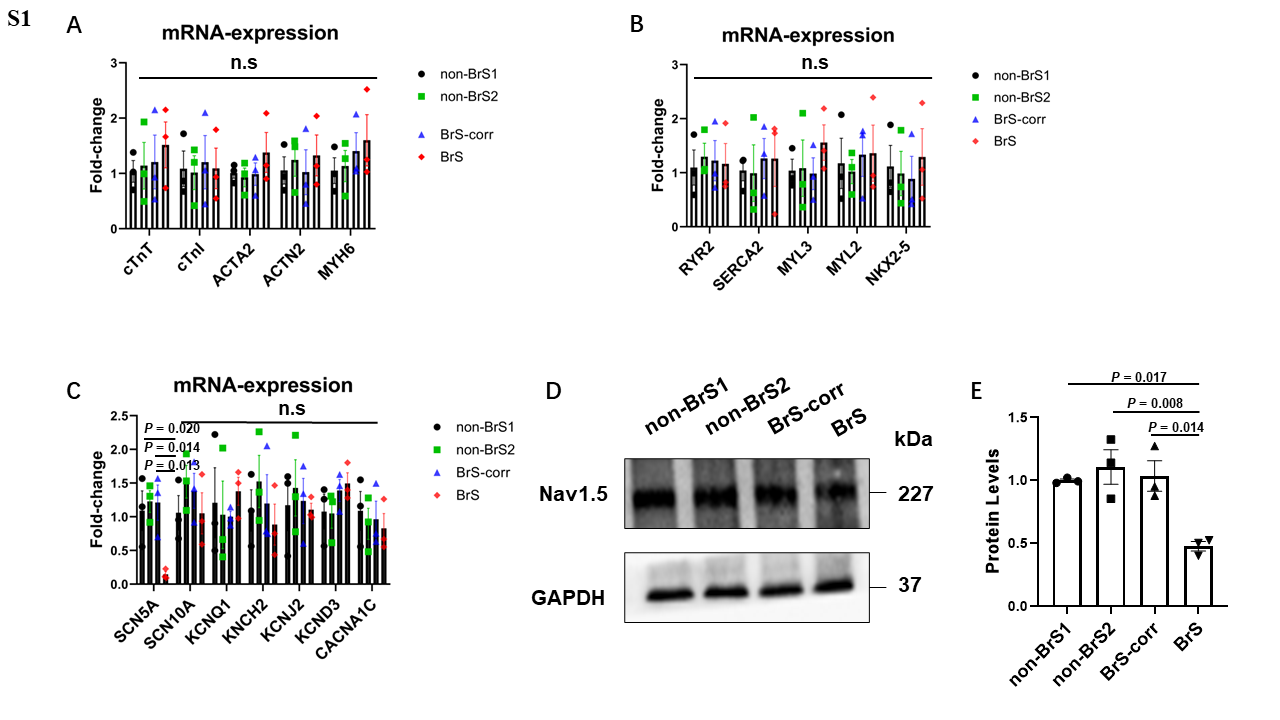

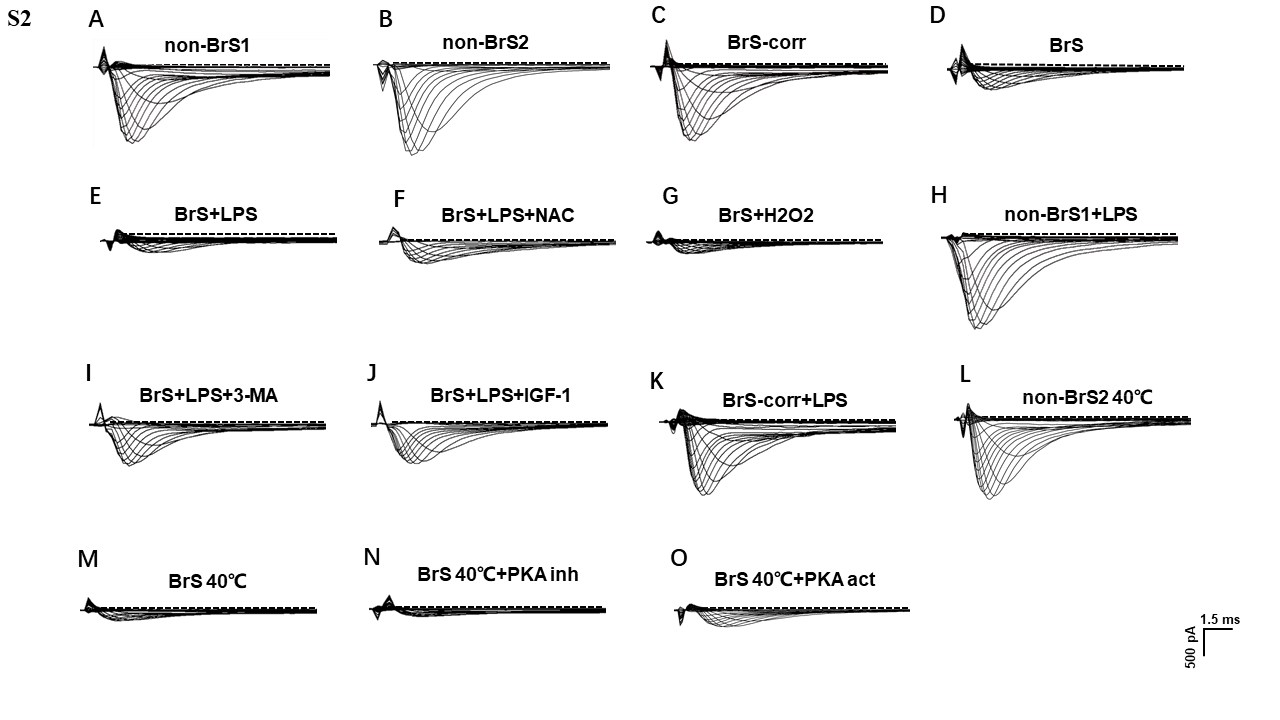
**
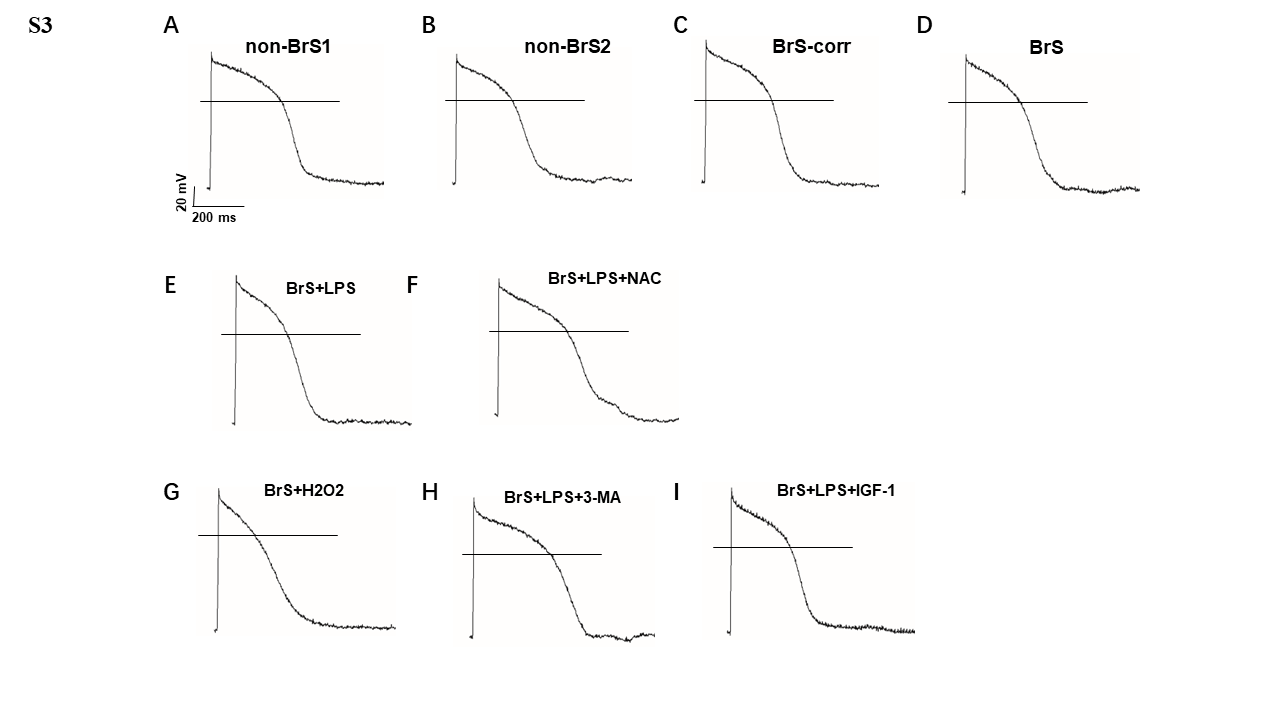
**
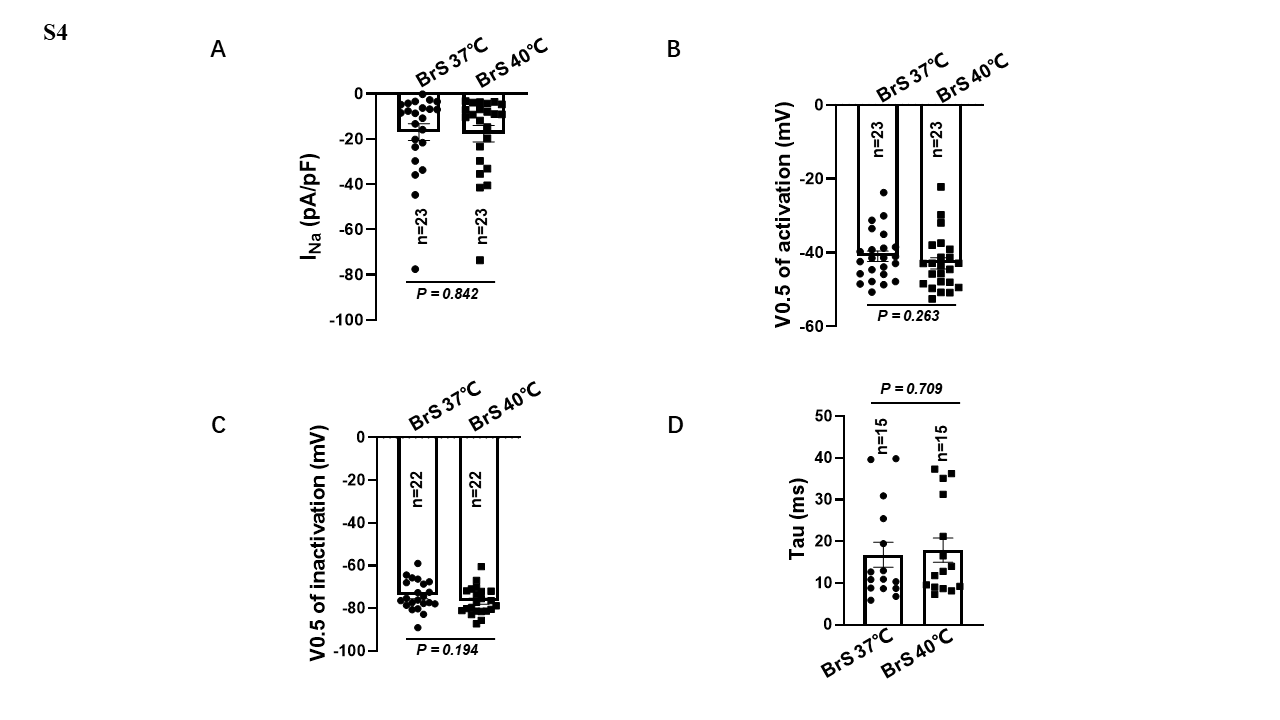

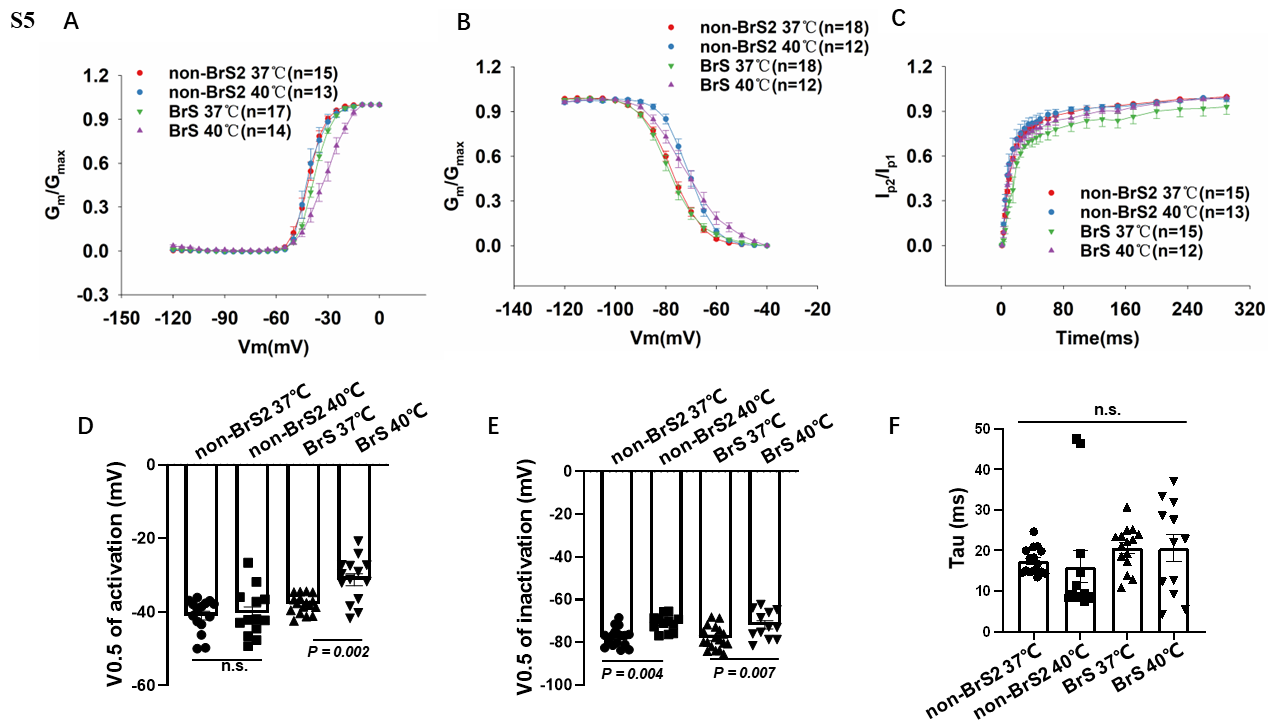

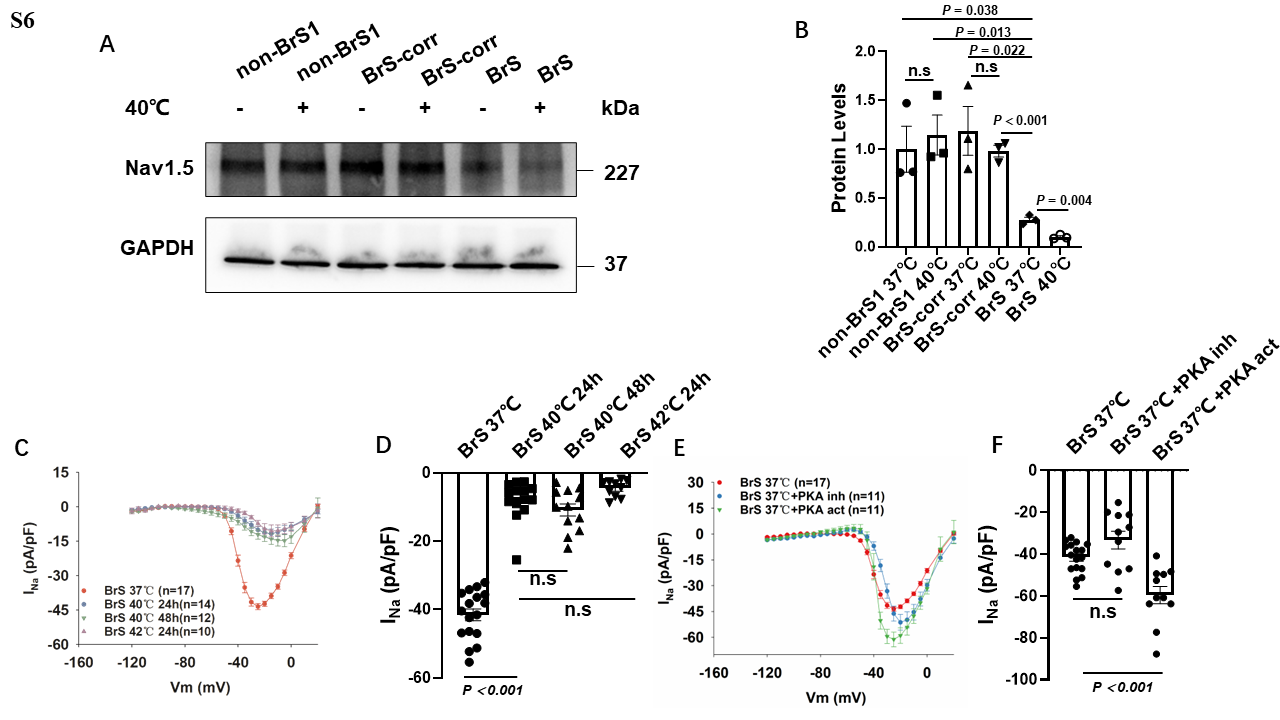
**
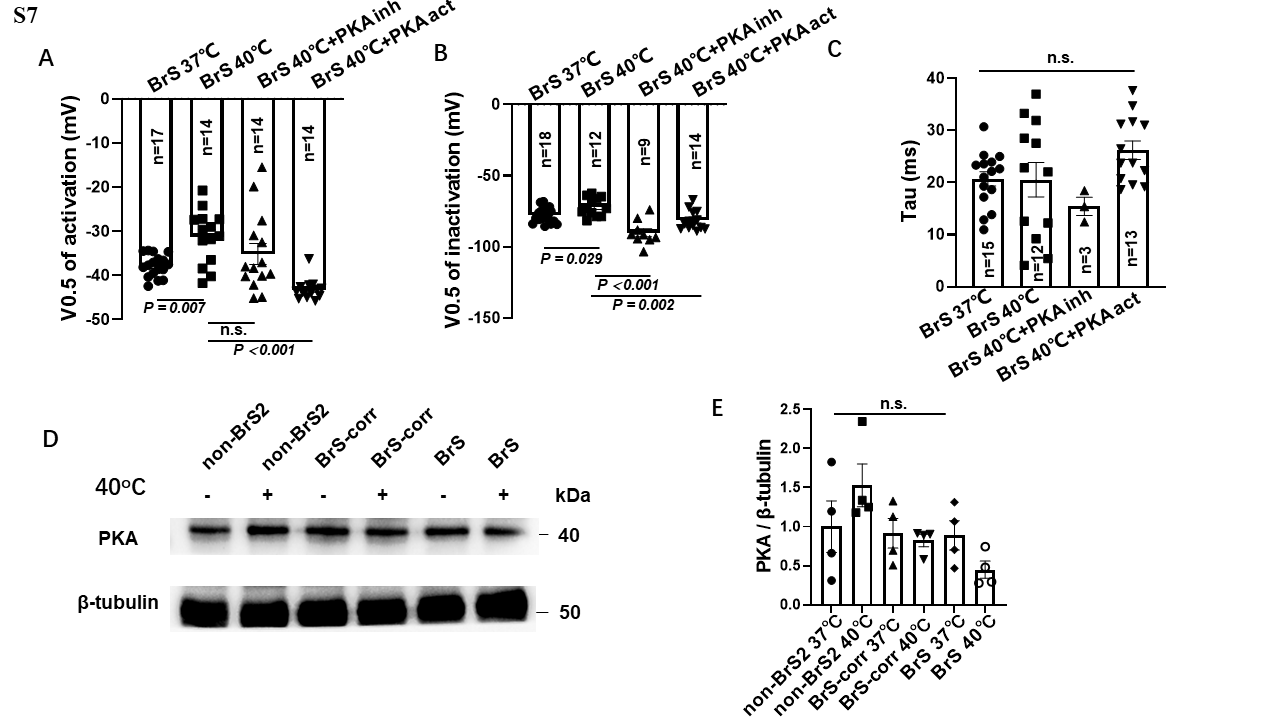
**
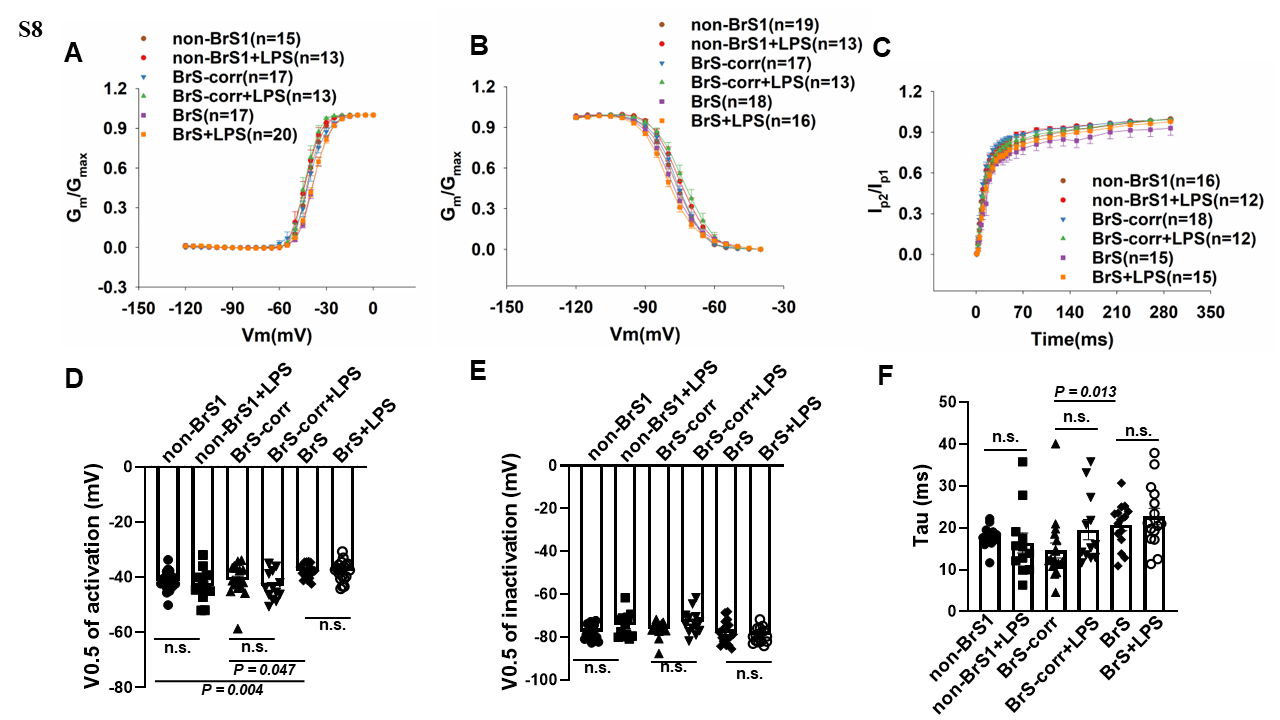

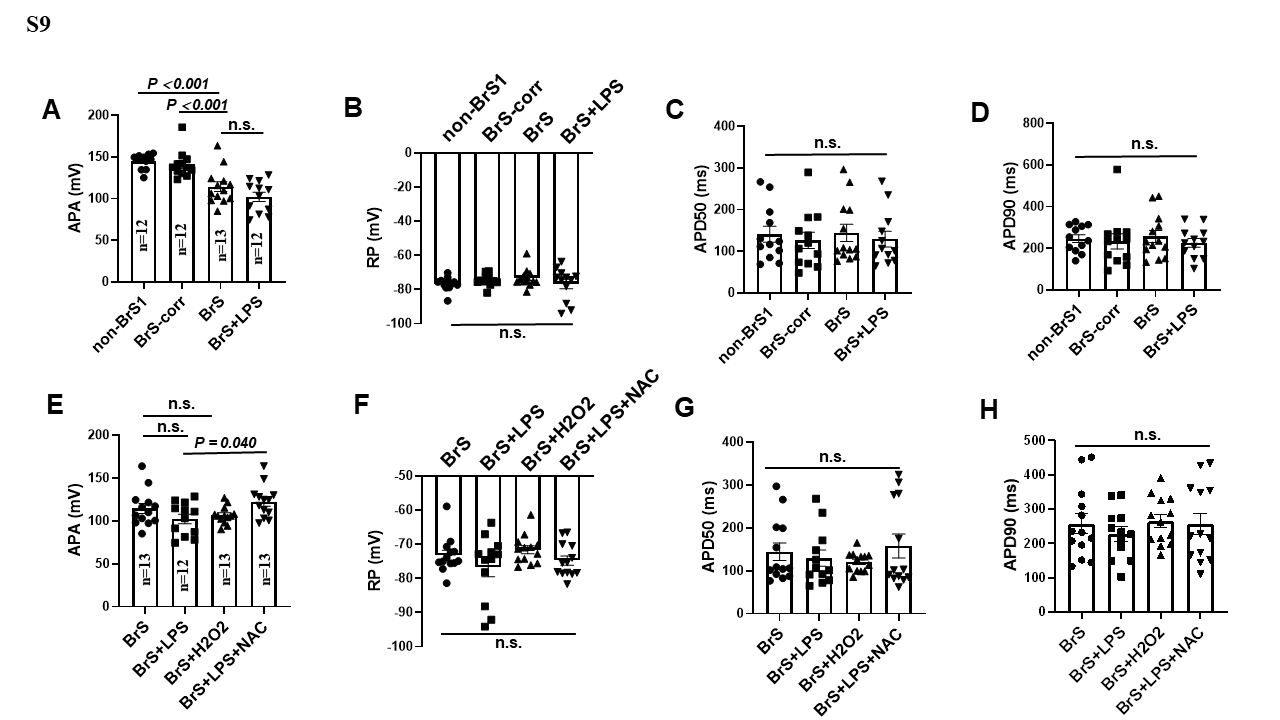

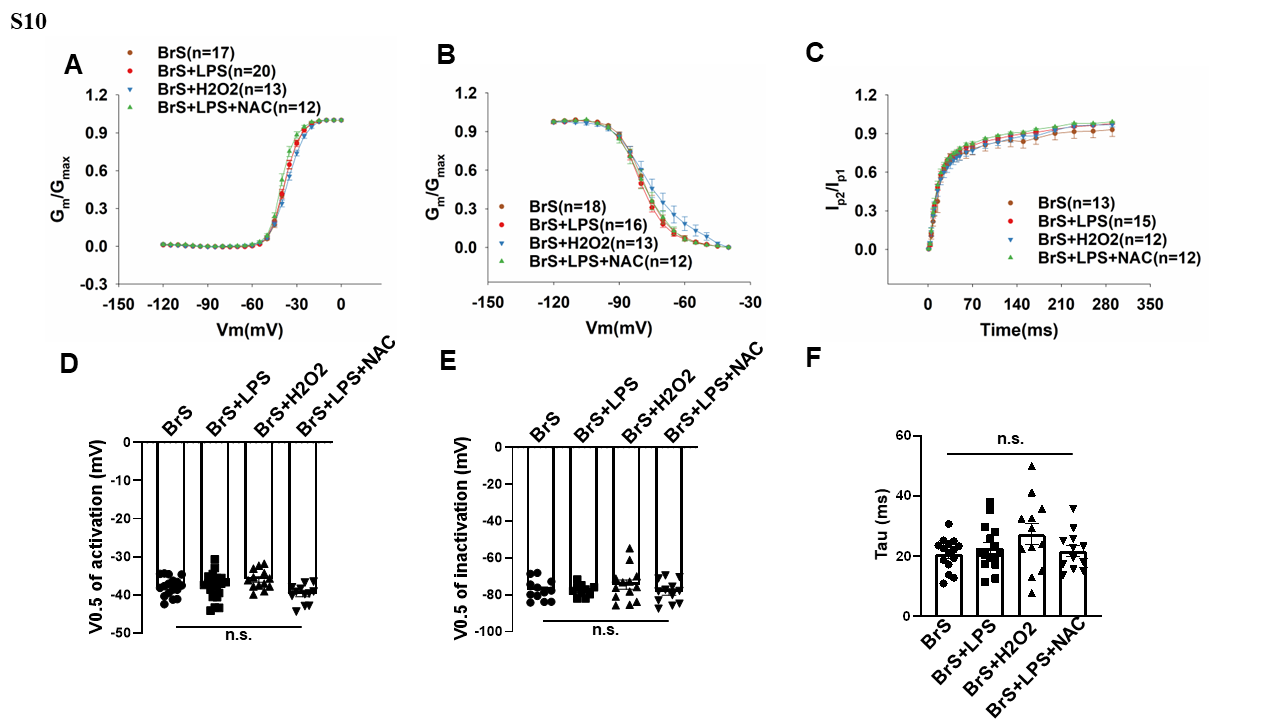

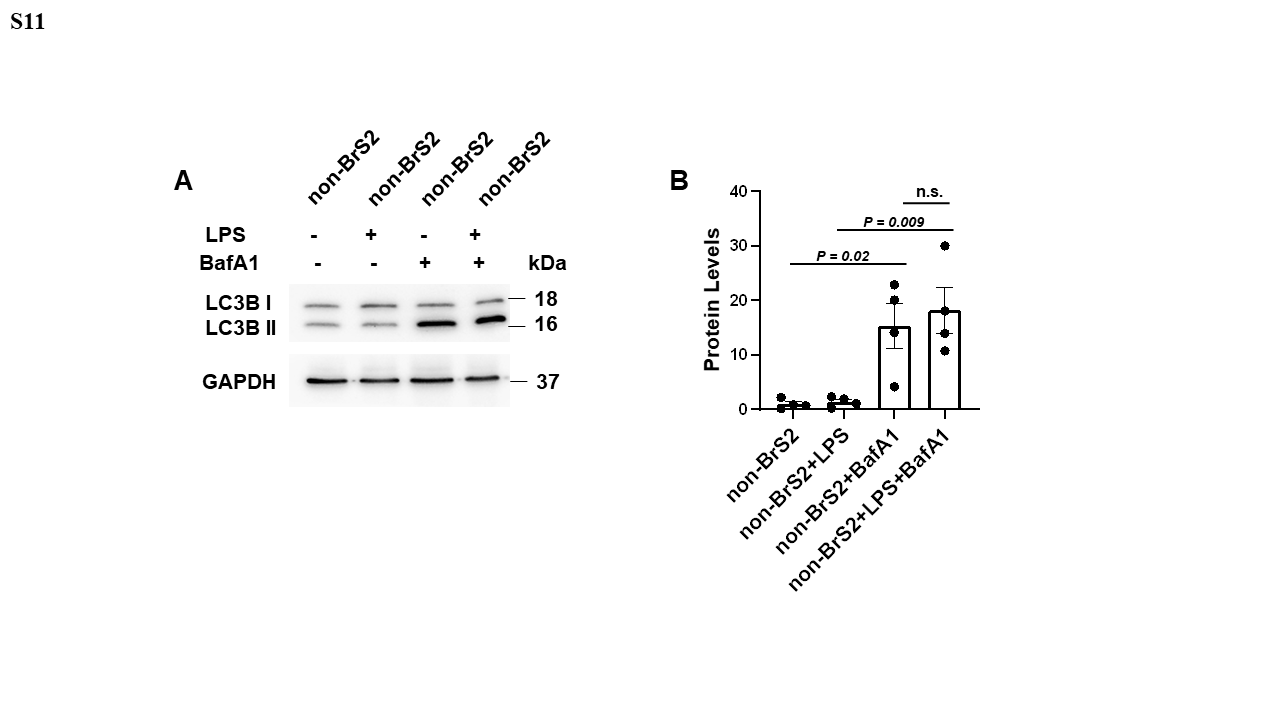

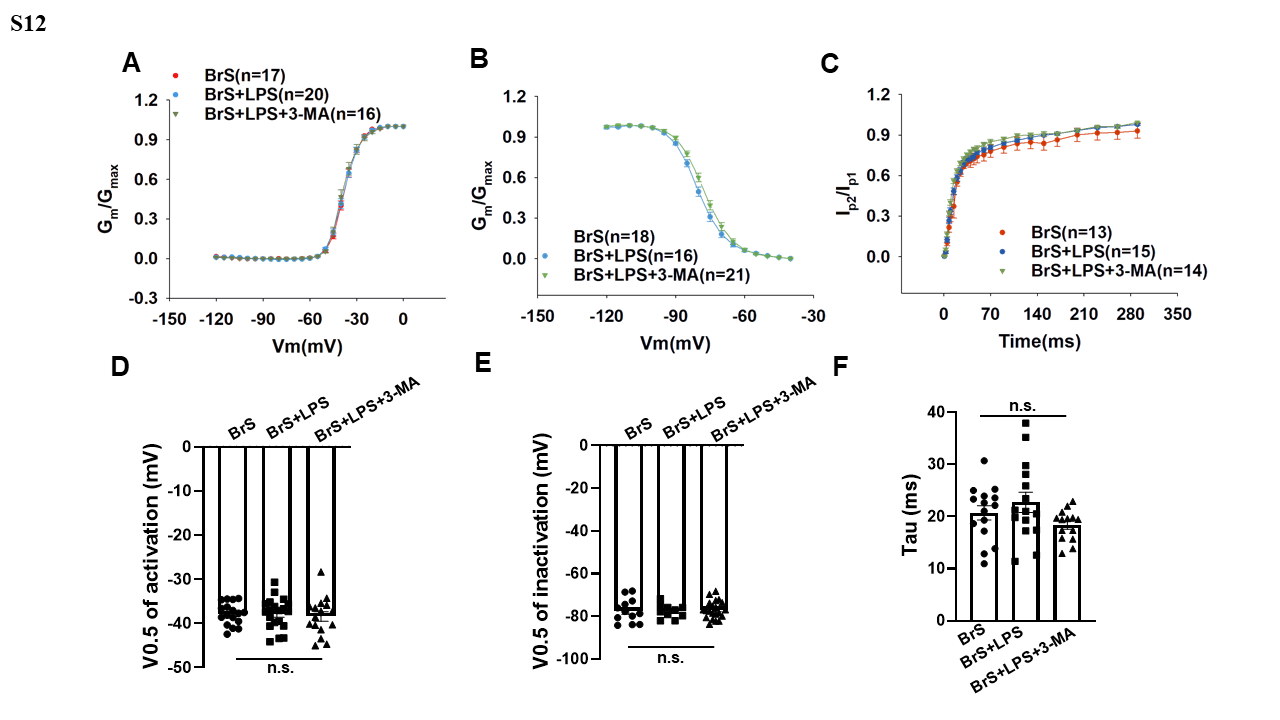

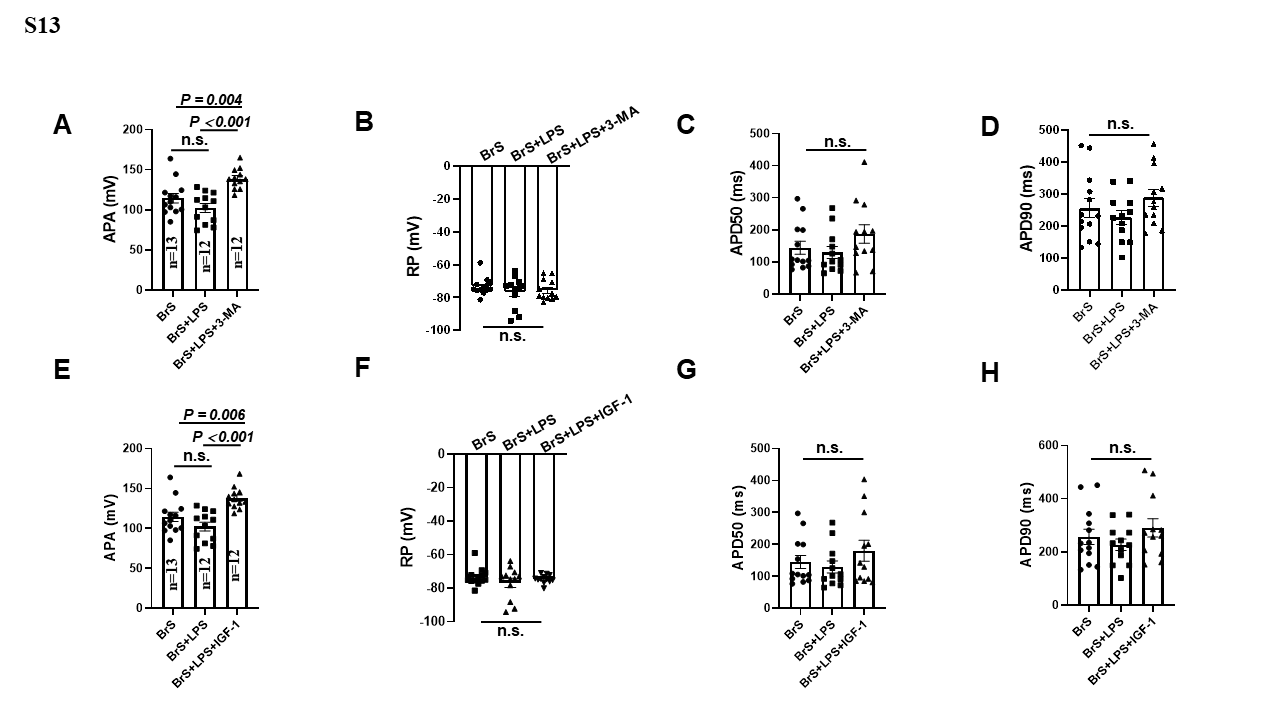

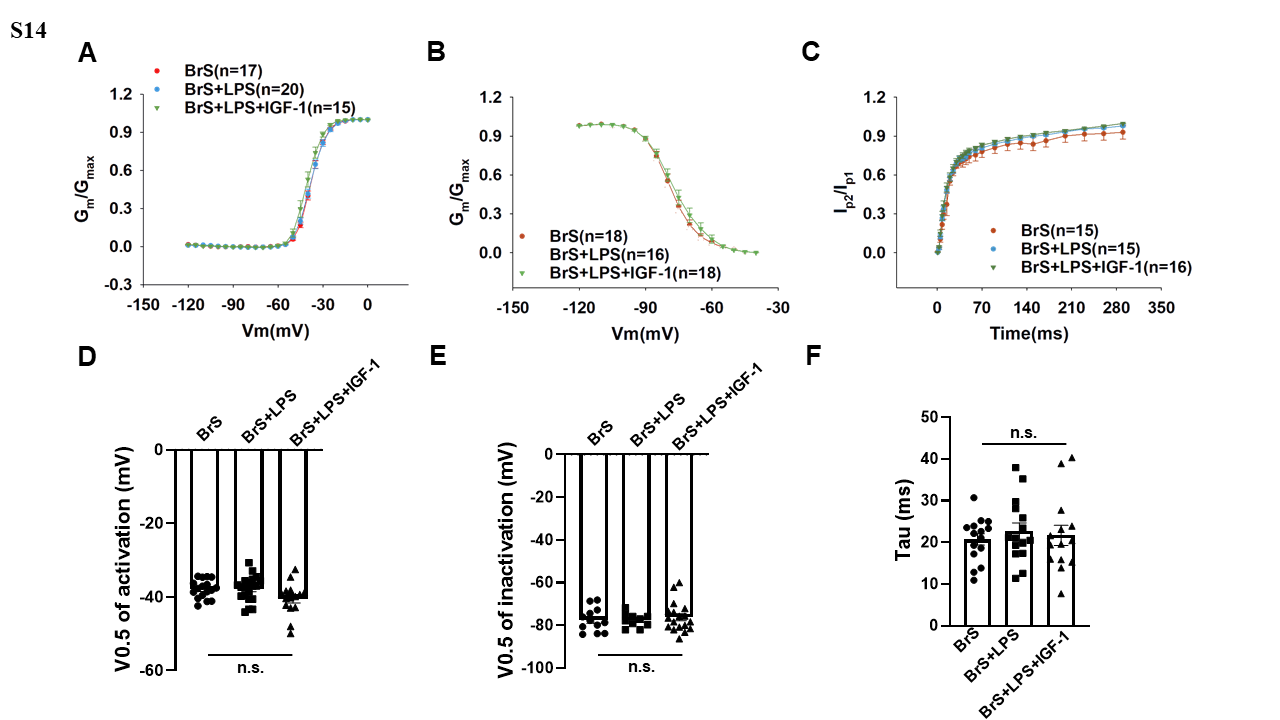

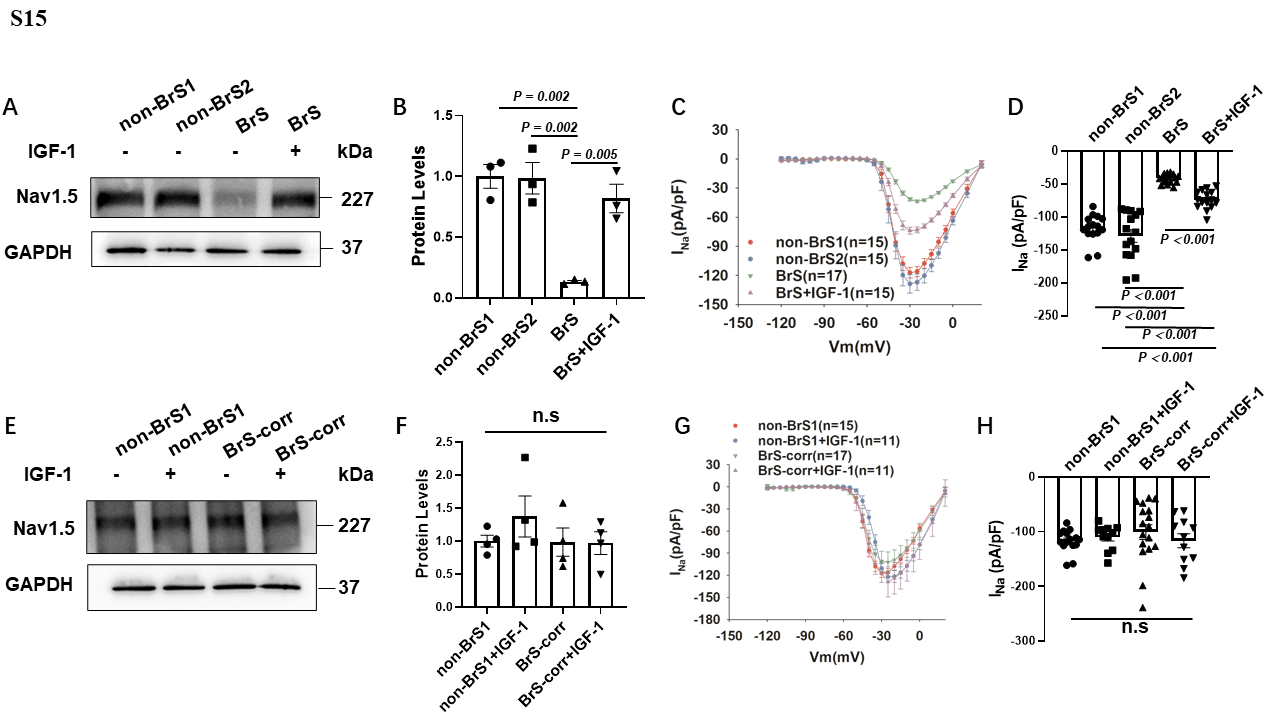

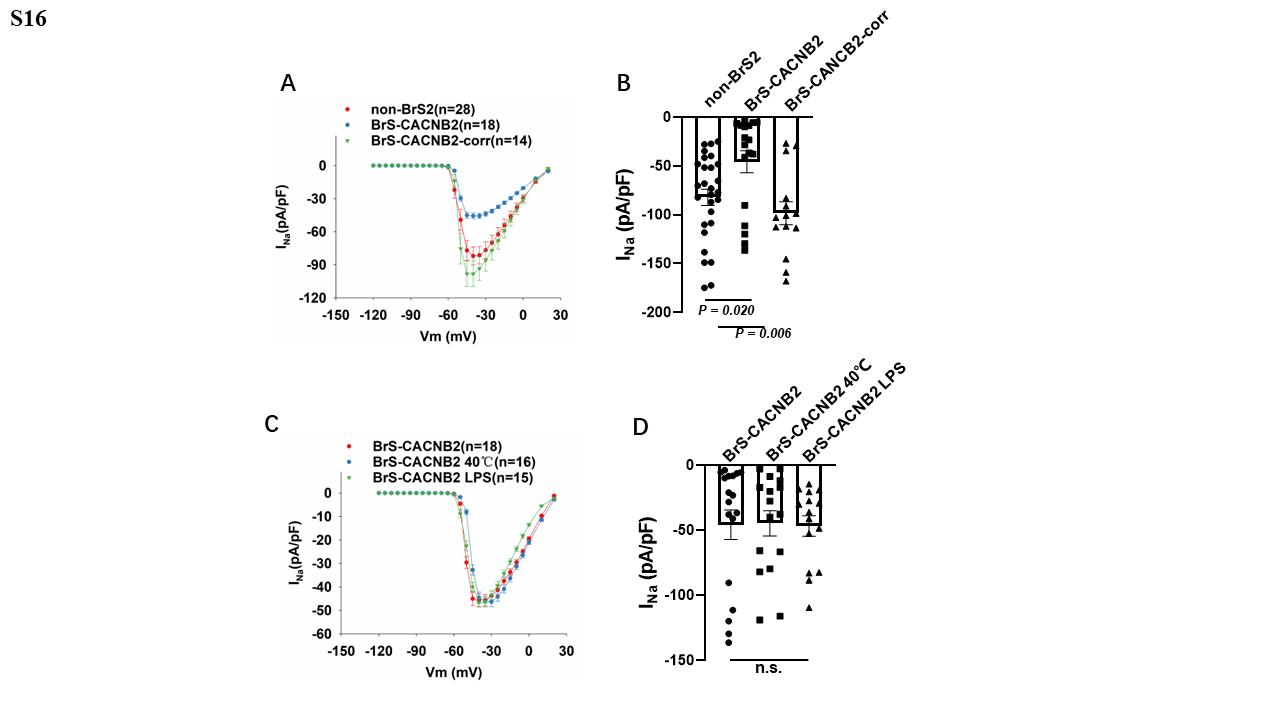
**
